# Nitric oxide precipitates catastrophic chromosome fragmentation by bolstering both hydrogen peroxide and Fe(II) Fenton reactants in *E. coli*

**DOI:** 10.1016/j.jbc.2022.101825

**Published:** 2022-03-11

**Authors:** Pooja Agashe, Andrei Kuzminov

**Affiliations:** Department of Microbiology, University of Illinois at Urbana-Champaign, Urbana, Illinois, USA

**Keywords:** oxidative stress, catalase, hydrogen peroxide, nitric oxide, iron metabolism, ferritin, respiration, double-strand DNA breaks, peroxynitrite, CCF, catastrophic chromosome fragmentation, CN, cyanide, DF, deferoxamine, *EPR*, electron paramagnetic resonance, Fre, flavin reductase, *H*_*2*_*O*_*2*_ or HP, hydrogen peroxide, IF-iron, intracellular free iron, NO, nitric oxide, ROS, reactive oxygen species, RNIs, reactive nitrogen intermediates

## Abstract

Immune cells kill invading microbes by producing reactive oxygen and nitrogen species, primarily hydrogen peroxide (H_2_O_2_) and nitric oxide (NO). We previously found that NO inhibits catalases in *Escherichia coli*, stabilizing H_2_O_2_ around treated cells and promoting catastrophic chromosome fragmentation *via* continuous Fenton reactions generating hydroxyl radicals. Indeed, H_2_O_2_-alone treatment kills catalase-deficient (*katEG*) mutants similar to H_2_O_2_+NO treatment. However, the Fenton reaction, in addition to H_2_O_2_, requires Fe(II), which H_2_O_2_ excess instantly converts into Fenton-inert Fe(III). For continuous Fenton when H_2_O_2_ is stable, a supply of reduced iron becomes necessary. We show here that this supply is ensured by Fe(II) recruitment from ferritins and Fe(III) reduction by flavin reductase. Our observations also concur with NO-mediated respiration inhibition that drives Fe(III) reduction. We modeled this NO-mediated inhibition *via* inactivation of *ndh* and *nuo* respiratory enzymes responsible for the step of NADH oxidation, which results in increased NADH pools driving flavin reduction. We found that, like the *katEG* mutant, the *ndh nuo* double mutant is similarly sensitive to H_2_O_2_-alone and H_2_O_2_+NO treatments. Moreover, the quadruple *katEG ndh nuo* mutant lacking both catalases and efficient respiration was rapidly killed by H_2_O_2_-alone, but this killing was delayed by NO, rather than potentiated by it. Taken together, we conclude that NO boosts the levels of both H_2_O_2_ and Fe(II) Fenton reactants, making continuous hydroxyl-radical production feasible and resulting in irreparable oxidative damage to the chromosome.

Mechanisms of bacterial killing by our immune cells are complicated and continue to attract experimental attention. Production of reactive oxygen species (ROS) superoxide and H_2_O_2_ ("HP" in figures) by the phagocyte NAD(P)H oxidase (phox) and reactive nitrogen intermediates (RNIs) by inducible nitric oxide synthase are important for killing endocytosed bacteria (1, 2, 3). Mice deficient in both gp91^*phox*^ and nitric oxide synthase are susceptible to spontaneous internal infections, demonstrating that ROS and RNI are important elements of a synergistic macrophage antimicrobial response (4).

While the charged superoxide cannot easily penetrate inside the cell, uncharged H_2_O_2_ does so by diffusion, where it reacts with intracellular free iron (IF-iron) *via* Fenton chemistry (5): **Fe(II) + H**_**2**_**O**_**2**_ **→ Fe(III) +** **·OH** **+ OH**^**–**^ to generate extremely reactive hydroxyl radicals, which can damage, among other molecules, chromosomal DNA (6, 7, 8). Due to the danger of damage to the genetic material, cells restrict Fenton chemistry using multiple systems (9, 10). Specifically in *Escherichia coli*, H_2_O_2_ is scavenged by catalases (11, 12) and peroxidases (13), while IF-iron is generally limited by the Fur regulon (14, 15) and is additionally sequestered in the presence of H_2_O_2_ (16, 17) (Fig. S1). In addition, the Fenton-induced DNA damage is repaired by base-excision repair and recombinational repair (18, 19, 20). These multiple systems in *E. coli* ensure that acute H_2_O_2_ doses up to 5 mM are only bacteriostatic in *E. coli*, although H_2_O_2_ concentrations ≥10 mM apparently self-potentiate to cause chromosomal fragmentation and loss of viability even in this quite resistant bacterium (7, 21, 22).

But there is complexity even in the mechanisms of *E. coli* killing by H_2_O_2_ (7, 23, 24). Generally, in cells with intact H_2_O_2_ scavenging, low millimolar H_2_O_2_ concentrations kill by mode-one; mode-one killing is blocked by iron chelators and affects mostly the DNA repair mutants, defining mode-one as IF-iron–dependent killing *via* DNA damage. When WT *E. coli* is killed by 10 mM H_2_O_2_, it is still mode-one (22). In contrast, higher concentrations of H_2_O_2_, starting with 20 to 25 mM in *E. coli*, kill by mode-two, which is insensitive of iron chelation, active metabolism, or of DNA repair capacity—defining mode-two as killing of unknown nature that does not depend on IF-iron or DNA damage. The additional distinction between the two H_2_O_2_ killing modes relevant for this work is that, while mode-one killing is potentiated by NO, mode-two killing is inhibited by NO treatment (22).

Perhaps the biggest paradox of H_2_O_2_ toxicity is that inside our immune cells, bacteria are presumed to be killed by H_2_O_2_ concentrations that are at least 1,000× lower than the H_2_O_2_ concentrations that start killing them in growing lab cultures. While direct electrochemical detection of ROS and RNI in phagosomes within macrophages with nanoelectrodes is still being developed (25) and the outside microelectrode measurements suggesting their extremely high (micromolar) concentrations (26, 27, 28) not being widely appreciated, researchers still operate with reasonable calculations that suggest H_2_O_2_ concentrations inside the macrophage and neutrophil phagosome are on the order of 10 μM (29, 30). At the same time, as mentioned above, the minimal H_2_O_2_ concentrations that kill WT *E. coli* in culture are 10 mM (7, 21, 22). To explain how such low H_2_O_2_ concentrations can be effective against bacteria in the phagosome, H_2_O_2_ was proposed and shown to be potentiated by other substances (reviewed in (31)), notably by nitric oxide (NO), abundantly produced by immune cells.

Indeed, NO and H_2_O_2_, when present simultaneously, kill WT *E. coli* at concentrations at which they are individually nonlethal (1, 32, 33) (Fig. 1*A*, left). We have previously demonstrated that the nature of this phenomenon is not simply redundancy of two toxic treatments overwhelming antitoxicity mechanisms of the cells but potentiation of H_2_O_2_ toxicity by NO to cause catastrophic chromosome fragmentation (CCF) (22) (Fig. 1*A*, right). NO, a radical species with one unpaired electron, can bind to other species with unpaired electrons, like transition metals (34), the intracellular ‘labile’ iron pool (35), iron in the regulatory iron–sulfur cluster-containing proteins (36), ferrous heme in ubiquinol oxidases, and ferric heme in catalases (37). Two general strategies around Fenton chemistry, by which NO could enhance the intracellular toxicity of H_2_O_2_, are as follows (Fig. S1): (1) increase in available Fe(II) and (2) stabilization of the intracellular H_2_O_2_.

**Figure 1:**
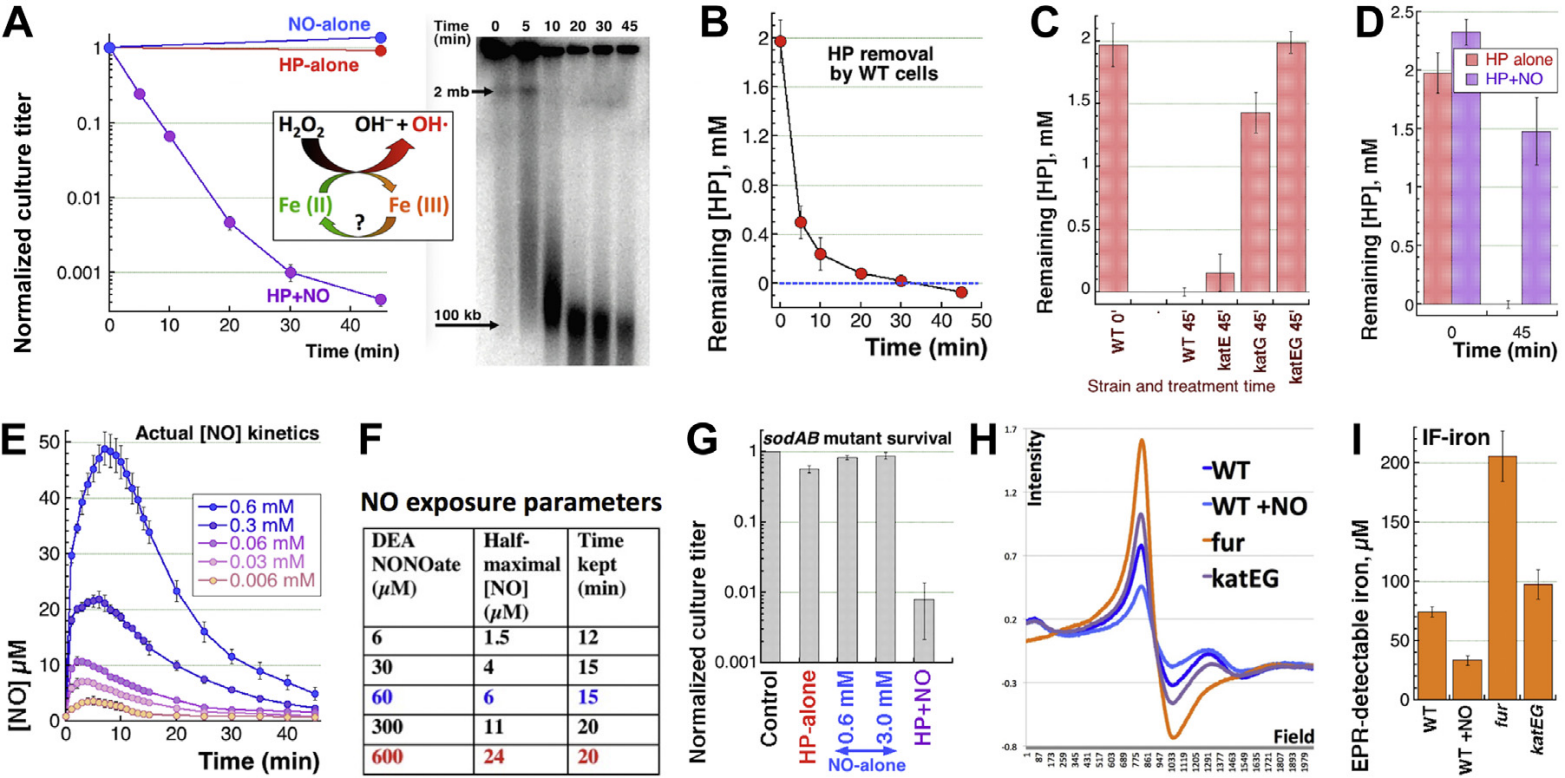
**Parameters of the experimental system: stability of hydrogen peroxide (HP in figures, H**_**2**_**O**_**2**_**in text), size, and duration of NO-bolus, the possibility of peroxynitrite, levels of IF-iron**. *A*, the previous results (22): (1) left, killing of WT cells by combined 2.5 mM H_2_O_2_ + 0.6 mM NO treatment, but not by individual treatments; (2) *right*, PFGE detection of catastrophic chromosome fragmentation in such H_2_O_2_+NO-treated cells; (3) inset, a scheme of the Fenton reaction, with the iron redox cycle shown separately and a possible NO participation in it as the question mark. *B*, a time course of H_2_O_2_ disappearance in WT cultures. Here and on, all values are means of three or more independent measurements ±SEM (that is, when error bars are almost touching, the two values are NOT different). When no error bars are visible, they are covered by the symbols. *C,* stability (survival) of H_2_O_2_ in cultures of catalase mutants. *D*, same but in WT cells treated with 0.6 mM DEA NONOate. *E,* evolution of actual NO concentrations in LB8 at 37 °C, starting from various indicated concentrations of DEA NONOate. *F,* the "NO-dose" that various DEA NONOate concentrations deliver: the half-maximal [NO] and the time period they are maintained in LB8 at 37 °C are determined from the graph in "E". *G,* the *sodAB* mutant sensitivity to 45 min treatments with H_2_O_2_-alone, NO-alone (0.6 mM and 3.0 mM), or H_2_O_2_+NO. *H,* EPR traces of Fe(III) in extracts of untreated WT cells, WT cells after 0.6 mM DEA NONOate treatment for 10 min, as well as *fur* and *katEG* mutants. *I,* concentration of IF-iron quantified from results like in "H". NO, nitric oxide; IF-iron, intracellular free iron; EPR, electron paramagnetic resonance.

The first strategy is confirmed in principle by the effect of iron chelators that completely block potentiated H_2_O_2_ toxicity (32, 38, 39). Additionally, Woodmansee and Imlay investigated a mechanism, dependent on iron, by which either cyanide (CN) or NO could potentiate H_2_O_2_ toxicity (38, 40). Both molecules complex the oxygen-binding heme of the terminal quinol oxidases (41), blocking respiration (42), which in the case of CN leads to accumulation of the reduced form of nicotinamide adenine dinucleotide, NADH (38); NO was proposed to do the same (40). Consequently, the enzyme flavin reductase (Fre) uses the increased NADH pool to reduce free flavins, which in turn reduce ferric iron to ferrous iron, which then becomes available for the Fenton reaction (38, 40). Since, in the presence of excessive H_2_O_2_, Fenton reaction rapidly turns all IF-iron from Fe(II) to Fe(III), NO should be able to potentiate Fenton by cycling Fe(III) back to Fe(II), continuously feeding Fenton reaction with the disappearing key ingredient, reduced iron (Fig. S1).

In another “iron-centric” model, we previously proposed that part of the CN potentiation of H_2_O_2_ toxicity was iron recruitment from cellular iron depots (21) and deposition of this iron directly onto DNA, causing chromosome fragmentation (20, 31). Bacterial cells store their iron in ferritins of two types: the regular size ferritins, FtnA and Bfr (the latter one equipped with internal heme), and the small ferritin, Dps (16, 17). In *E. coli*, the FtnA ferritin functions like a regular iron depot, taking in excess iron and releasing it back when needed; the function of Bfr is less clear (16, 17, 43). Ferritins can release ferrous iron in response to reductants, such as thiols, ascorbate, or flavins (44, 45, 46). Both ferritins and bacterioferritins have been shown to release iron by flavin-dependent and flavin-independent ferric reductases (15, 47). In contrast, Dps appears to function as a terminal iron repository in the presence of H_2_O_2_, as the Dps-sequestered iron can be subsequently released only by Dps protein degradation (48). Using corresponding single mutants and H_2_O_2_ potentiation by CN, we have previously shown (21) that Dps is important for protecting chromosomal DNA, while FtnA serves as a source of iron for the H_2_O_2_+CN attack on the chromosomal DNA; inactivation of Bfr had no effect.

The above studies demonstrated that manipulating the intracellular iron causes synergistic toxicity with H_2_O_2_ *via* increasing DNA damage (20, 21, 38, 40). Curiously, catalase inhibition by CN or NO, even though long-known *in vitro* (49, 50, 51, 52), was for various reasons not considered as a pathway of potentiation of H_2_O_2_ toxicity *in vivo* (21, 38, 40). It was even argued that H_2_O_2_ detoxification was prioritized over NO detoxification when both agents were present together (in much reduced, close-to-physiological concentrations) (53). In contrast, our recent study of the H_2_O_2_+NO toxicity found that NO inhibits H_2_O_2_ scavenging, by binding and inhibiting the heme-containing catalases (Fig. S1), to stabilize effective concentrations of H_2_O_2_ inside the treated cells, causing lethal densities of double-strand DNA breaks (22). We also found that in a catalase-deficient mutant, H_2_O_2_-alone exerts mode-two killing at H_2_O_2_ concentrations static for WT cells, while NO treatment blocks unknown targets of mode-two killing, offering temporary protection from lethal H_2_O_2_ concentrations (22).

In this study, we wanted to determine the contribution of various intracellular targets of NO potentiation to the overall H_2_O_2_+NO killing. Is the catalase inhibition by NO and resulting H_2_O_2_ stability the cause of death or does it simply correlate with a concurrent respiration inhibition in WT cells? Another objective was to find additional cellular targets of NO, if they exist, that could potentiate H_2_O_2_ toxicity. Also, there could be mutants/conditions, in which NO would alleviate H_2_O_2_ lethality, as described for other organisms (54, 55) and in our previous study (22). In short, this article addresses how NO affects the "iron" side of Fenton's reactants (Fig. 1*A*, inset, Fig. S1).

## Results

### Parameters of the H_2_O_2_+NO treatment

To study NO potentiation of H_2_O_2_ toxicity, we treat *E. coli* cultures, growing in LB8 (LB buffered with 50 mM Tris HCl pH = 8.0) with 2.5 mM H_2_O_2_ and 0.6 mM DEA NONOate (H_2_O_2_ + NO henceforth), two treatments that are bacteriostatic by themselves yet kill within minutes in combination, by precipitating CCF (Fig. 1*A*) (22). As previously measured by a spectrophotometric assay (22), and here by an H_2_O_2_ electrode, 2.5 mM H_2_O_2_ is completely degraded by WT cells in 20 min (Fig. 1*B*) but could be stabilized by genetic inactivation of both catalases (Fig. 1*C*). The *katE* or *katG* single mutants also show reduced capacity to degrade H_2_O_2_ (Fig. 1*C*); however, unlike the sensitive *katEG* double mutant, both single mutants are resistant to H_2_O_2_-alone treatment (22), showing that their residual scavenging capacity is adequate for intracellular protection against H_2_O_2_-alone.

Besides catalase inactivation, H_2_O_2_ in *E. coli* is similarly stabilized by NO (Figs. 1*D* and S2) (22). NO in our treatment is produced by decomposition of NO-donor DEA NONOate; its slower release because of the higher pH of our LB8 explains the 2 to 6 min rise to a concave plateau around the maximal concentration, followed by 10 to 15 min decline to the half-maximal concentration transitioning into a decomposition tail (Fig. 1*E*). The unexpectedly rapid decline is because NO gas readily escapes from aqueous solutions, unless they are in bioreactors (53) or overlaid with mineral oil (56). We characterize NO exposure of the cultures at any given concentration of DEA NONOate with two parameters derived from the evolution curves of Figure 1*E*: the half-maximal [NO] and the period during which [NO] was above the half-maximal concentration (Fig. 1*F*). For example, starting with 600 μM DEA NONOate, NO exposure is 24 μM x 20 min [this exposure inhibits catalases in Fig. 1*D* by ∼95% (and see below)], while starting with 60 μM DEA NONOate, NO exposure is reduced to 6 μM x 15 min, and the catalase inhibition is only ∼50% (see below). NONOate donors are known to yield actual NO concentrations much lower than the initial donor concentrations (22, 53, 57, 58). Henceforth, we will abbreviate DEA NONOate as "NO".

Because H_2_O_2_ is produced by phagosomes indirectly, *via* production of superoxide (59), there is a possibility that the real bactericidal species in the presence of NO is peroxynitrite (3, 60), formation of which by the combination of superoxide and NO is indeed limited only by diffusion (61). We tested this idea in superoxide dismutase–deficient mutant *sodAB*, which accumulates significant levels of the intracellular superoxide (62) and therefore, unlike the WT, should be killed by NO-alone treatment and should be additionally sensitive to H_2_O_2_+NO treatment, if peroxynitrite is indeed so toxic. Yet, the *sodAB* mutant shows no sensitivity to either the standard NO dose or even 5× higher (3 mM) NO-alone treatment (Fig. 1*G*). Additionally, the mutant is significantly less sensitive than WT to H_2_O_2_+NO treatment (compare Fig. 1*A* *versus* G), which we also observed before for a similar H_2_O_2_+CN treatment (21). It seems as if peroxynitrite, even if it could form in the cytoplasm, does not pose the same threat as the H_2_O_2_+NO combination.

Finally, since we addressed the role of iron in this work, we used electron paramagnetic resonance (EPR) to measure the concentration of IF-iron in our cells (Figs. 1, *H* and *I* and S3). WT cells provide the baseline of ∼75 μM of IF-iron, which is higher than 20 to 30 μM found in *E. coli* grown in minimal media (38, 63) but is within the range reported for *E. coli* grown in LB (64, 65). At the same time, the *fur* mutant, deficient in the cytoplasmic iron regulation, accumulates up to 200 μM IF-iron (Fig. 1, *H* and *I*), which is also well within the range for this mutant when grown in LB (64, 65). Therefore, if in a given mutant, the level of IF-iron is the same as in the WT before starting the treatment, it is safe to interpret the change in the mutant's sensitivity to H_2_O_2_+NO in terms of either additional iron recruitment/reduction (cycling) by NO or as NO-inhibition of catalases. For example, the levels of IF-iron remain roughly the same in the *katEG* mutant (Fig. 1, *H* and *I*), showing that catalase inactivation does not upset iron homeostasis in *E. coli* (unlike, for example, does the inactivation of superoxide dismutases (66)).

### The *fur* mutant shows effects of excess IF-iron

**Figure 2:**
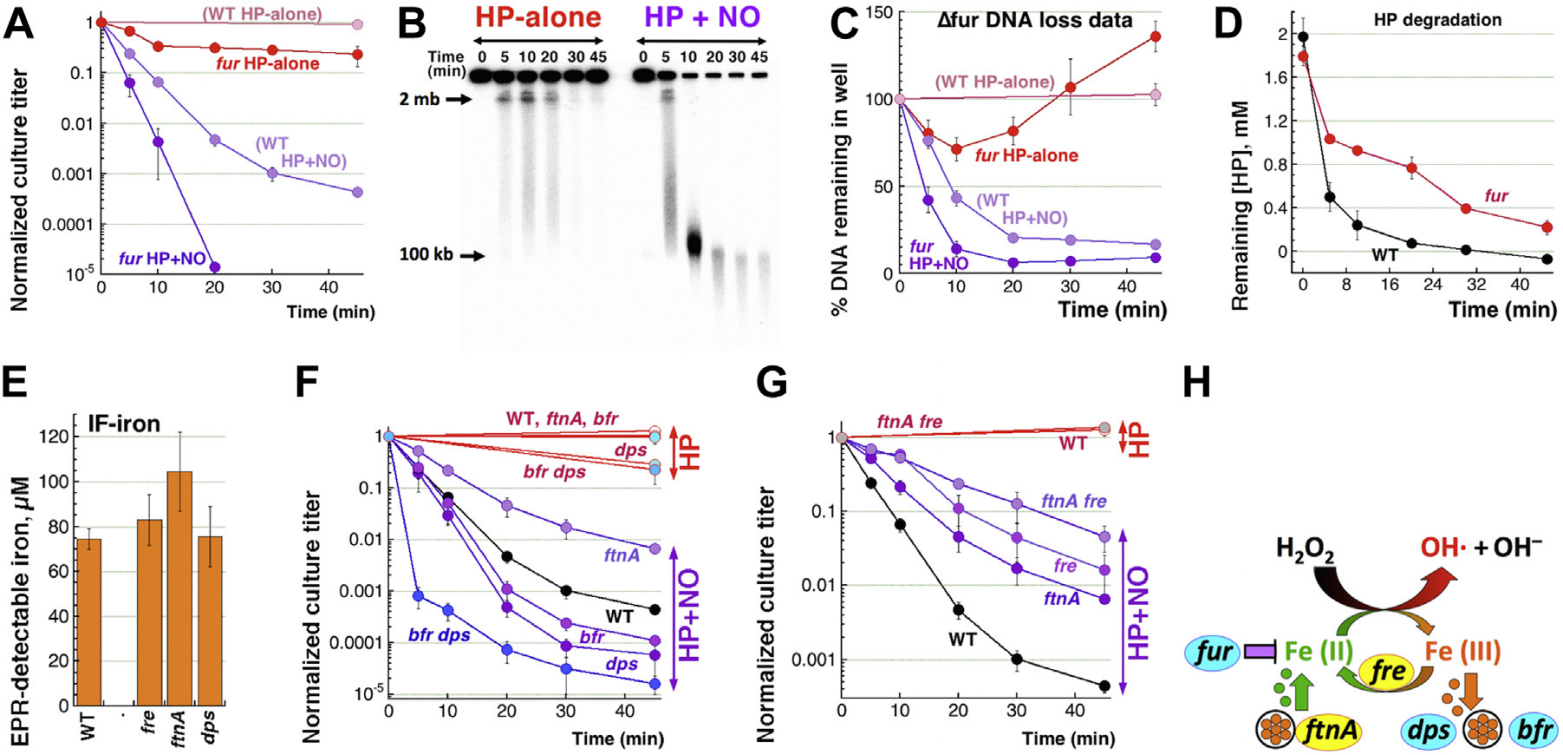
**The importance of the iron side of the Fenton reactants.***A*, H_2_O_2_-alone and H_2_O_2_+NO sensitivity of *fur* mutants, unregulated for the intracellular free iron. *B*, a representative gel illustrating chromosome fragmentation patterns caused by H_2_O_2_-alone or H_2_O_2_+NO treatments in the *fur* mutant. *C,* quantification of the chromosomal DNA loss in the *fur* mutant in response to H_2_O_2_-alone or H_2_O_2_+NO treatments, from several gels like in "B". *D,* H_2_O_2_ is scavenged slower by the *fur* mutant. *E,* concentration of IF-iron in the indicated mutants compared to WT. *F,* H_2_O_2_-alone and H_2_O_2_+NO sensitivity of *ftnA*, *bfr,* and *dps* mutants in the three ferritins of *E. coli* cells. *G,* H_2_O_2_-alone and H_2_O_2_+NO sensitivity of the *fre* single and *ftnA fre* double mutants. *H,* iron amount, recruitment, and cycling are important for NO-potentiated H_2_O_2_ toxicity. In this elaborated scheme of the Fenton reaction, in *cyan* are the functions countering H_2_O_2_+NO toxicity, while in *yellow* are the functions aiding H_2_O_2_+NO toxicity—therefore, candidates for NO potentiation. EPR, electron paramagnetic resonance; H_2_O_2_/HP, hydrogen peroxide; IF-iron, intracellular free iron; NO, nitric oxide.

Previously, we showed that inactivation of catalases is necessary to explain NO potentiation of H_2_O_2_ toxicity (22), but is it sufficient (that is, the only way NO could act)? If stabilizing H_2_O_2_ *via* catalase inactivation is the only route of NO potentiation of H_2_O_2_ toxicity, then manipulating the other Fenton reactant, the IF-iron, should have no effect on killing by H_2_O_2_+NO. The levels of IF-iron are increased in the *fur* mutant (66); in our growth conditions, *fur* mutants have ∼2.5× more IF-iron than WT cells do (Fig. 1*I*). This increased iron makes the *fur* mutant only slightly sensitive to H_2_O_2_-alone treatment, yet significantly more sensitive than WT to H_2_O_2_+NO treatment (Fig. 2*A*). Thus, having more IF-iron available for Fenton makes cells vulnerable to H_2_O_2_, regardless of the presence or absence of NO.

The increased IF-iron in the *fur* mutant also accelerates the rate of chromosome fragmentation by H_2_O_2_+NO, leading to close to background levels of the remaining chromosomal DNA by 20 min of the treatment (Fig. 2, *B* and *C*). In other words, intact chromosomal DNA all but disappears in the *fur* mutant treated with H_2_O_2_+NO. H_2_O_2_-alone treatment is even more interesting; coincident with the small drop in survival (Fig. 2*A*), there is a 25% loss of the chromosomal DNA in the first 10 min, which is then followed by a surprising resumption of label incorporation after 20 min (Fig. 2, *B* and *C*). This fast ‘DNA recovery’ could be due to a faster scavenging of H_2_O_2_ in the *fur* mutant; however, contrary to this expectation, we found that the *fur* mutant actually scavenges H_2_O_2_ slower than WT cells (Fig. 2*D*), so that ∼1/3 of the original amount still remains after 20 min, at the time when essentially no H_2_O_2_ is detected in the medium of WT cells. A H_2_O_2_ scavenging defect has been reported previously in a *fur* mutant, wherein the sensitivity to H_2_O_2_ was attributed to low catalase activity rather than iron overload (67). Therefore, the nature of the resumption in chromosomal label incorporation in H_2_O_2_-alone–treated *fur* mutant cells, in spite of the higher [H_2_O_2_], remains unclear and likely reflects the significant changes in expression profile in this mutant (68) accelerating restart of a process inactivated by oxidative stress.

### The *ftnA* and *fre* defects alleviate H_2_O_2_+NO toxicity, while dps and bfr defects potentiate it

Since having more iron in the cytoplasm sensitizes cells to H_2_O_2_ exposure, we tested more mutants with defects in iron handling. Defects in the iron depot proteins ferritins FtnA and Dps do not affect the level of IF-iron in a statistically significant way (Fig. 2*E*). However, regular ferritins (homologs of FtnA of *E. coli*) are known to release iron, if stimulated by chemicals like NO or CN (31, 45). In contrast, the small ferritin Dps sequesters IF-iron in the presence of H_2_O_2_ (69). While *ftnA* and *bfr* mutants are not sensitive to H_2_O_2_-alone and *dps* mutant is only slightly sensitive, they do show differences from WT in their sensitivity to H_2_O_2_+NO (Fig. 2*F*). In particular, the *ftnA* inactivation alleviates H_2_O_2_+NO lethality (Fig. 2*F*), suggesting that FtnA depots provide a source of iron for the Fenton chemistry inside the cell. Contrary to this general idea and at the same time confirming the previous report (Woodmansee and Imlay 2003), we detected less IF-iron in WT cells treated with NO (Fig. 1, *H* and *I*). It could be that, in the presence of NO, the ferritin-released iron is immediately bound by DNA or by other big molecules, like stable RNA, to become undetectable by EPR. We have observed something like this before *in vitro* with CN-complexed IF-iron, when the added plasmid DNA effectively sequestered iron away from CN complexes (21).

In contrast to the ferritin deficiency, both the *bfr* and *dps* inactivation aggravate sensitivity to H_2_O_2_+NO treatment (Fig. 2*F*). Moreover, the double *bfr dps* mutant shows extreme sensitivity (Fig. 2*F*), suggesting that Dps and Bfr ferritins are redundant, and both are effective in sequestering iron when H_2_O_2_ is around. Thus, the H_2_O_2_+NO sensitivity of iron depot mutant confirms that in addition to H_2_O_2_ stabilization, NO potentiation does have a significant "iron dimension" to it, either releasing additional iron from iron depots FtnA or competing with IF-iron sequestration into Dps and Bfr.

Increased or decreased H_2_O_2_+NO sensitivity of ferritin mutants suggests a source of additional iron (FtnA) for promoting Fenton, but what is the mechanism of iron recruitment and keeping it reduced in the presence of H_2_O_2_? To state the problem differently, IF-iron will catalyze Fenton upon encounter with H_2_O_2_, but does it take multiple rounds of redox iron cycling to damage the chromosome beyond repair? To distinguish between the limited *versus* continuous Fenton, we inactivated the main Fe(III) → Fe(II) reduction enzyme, Fre. As the major siderophore reductase of *E. coli*, Fre catalyzes Fe(III) reduction to Fe(II) during iron acquisition from the environment (44). However, this metabolic activity becomes harmful during H_2_O_2_ exposure: by reducing the oxidized Fe(III) back to Fe(II), Fre keeps Fenton reaction going as long as reducing equivalents and H_2_O_2_ are available (38).

Indeed, the *fre* mutant shows resistance to H_2_O_2_+NO (Fig. 2*G*) (40), similar to its resistance to H_2_O_2_+CN (21, 38). The *fre* mutant has the same level of IF-iron as WT cells (Fig. 2*E*), indicating that (1) its decreased sensitivity to H_2_O_2_+NO is not due to a reduced levels of IF-iron and (2) no matter what the contribution of iron recruitment, continuous Fe(III) reduction back to Fenton-reactive Fe(II) is important for H_2_O_2_ toxicity in WT cells. Moreover, iron reduction by Fre seems to work in the same pathway as iron release from FtnA, as the H_2_O_2_+NO survival of the double *ftnA fre* mutant is not significantly different from those of single mutants (Fig. 2*G*). Overall, we conclude that the NO potentiation of the iron side of Fenton reaction significantly contributes to the overall lethality of the double treatment (Fig. 2*H*), whereas its complexity warrants further exploration, especially in comparison with the relatively straightforward NO stabilization of H_2_O_2_ *via* inhibition of catalases.

### Decreased iron reduction delays H_2_O_2_ toxicity in the *katEG* mutants

To probe the role of iron in the NO potentiation of H_2_O_2_ killing, H_2_O_2_-alone and H_2_O_2_+NO–treated cultures need to be compared. However, the two treatments are different in an important aspect: H_2_O_2_-alone is rapidly scavenged by catalases (Fig. 1*B*), while in the combined treatment, H_2_O_2_ is stable due to catalase inhibition by NO (Fig. 1*D*). To make H_2_O_2_-alone and H_2_O_2_+NO treatments comparable, we used the *katEG* catalase-deficient background to ensure stability of H_2_O_2_ concentrations for the duration of experiment (Fig. 1*C*). Indeed, the *katEG* double mutant is equally sensitive to H_2_O_2_-alone and H_2_O_2_+NO treatments (22); at the same time, since the *katEG* mutant has IF-iron levels comparable to that of WT cells (Fig. 1*H*), this sensitivity to H_2_O_2_-alone can be mostly attributed to the unscavenged H_2_O_2_.

**Figure 3:**
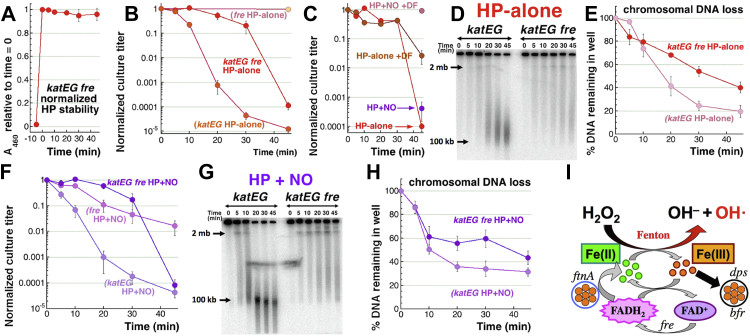
**The phenotypes of the triple *katEG fre* mutant emphasize the importance of iron cycling.***A*, H_2_O_2_ stability in the cultures of the *katEG fre* mutant using colorimetric detection of *o*-dianisidine by horseradish peroxidase. *B*, H_2_O_2_-alone sensitivity of the *katEG fre* triple mutant. *C,* the effect of deferoxamine (DF) on the *katEG fre* mutant sensitivity to H_2_O_2_-alone *versus* H_2_O_2_+NO. *D,* a representative gel to compare chromosome fragmentation patterns in the *katEG* double mutant *versus katEG fre* triple mutant, caused by H_2_O_2_-alone treatment. *E,* quantification of the chromosomal DNA loss in the *katEG fre* triple mutant in response to H_2_O_2_-alone treatment, from several gels like in "D". *F,* H_2_O_2_+NO sensitivity of the *katEG fre* triple mutant. *G,* a representative gel to compare chromosome fragmentation patterns in the *katEG* double mutant *versus katEG fre* triple mutant, caused by H_2_O_2_+NO treatment. *H,* quantification of the chromosomal DNA loss in the *katEG fre* triple mutant in response to H_2_O_2_+NO treatment, from several gels like in "G". *I*, the iron side of Fenton reactants, driven by FtnA and Fre, ensures continuous flow of Fe(II), but in the presence of H_2_O_2_, the IF-iron pool is also drained by Dps and Bfr. Fre, flavin reductase; H_2_O_2_/HP, hydrogen peroxide; IF-iron, intracellular free iron; NO, nitric oxide.

Our first question was whether the IF-iron levels by themselves are lethal when H_2_O_2_ is stable. To address it, we measured H_2_O_2_-alone and H_2_O_2_+NO sensitivity of the *katEG fre* mutant, whose catalase deficiency makes H_2_O_2_ stable (Fig. 3*A*), while the *fre* defect in iron reduction should restrict repeated cycles of Fenton. When confronted with H_2_O_2_-alone, the *katEG fre* triple mutant showed a hybrid sensitivity pattern: until 30 min of the treatment, the mutant was almost as resistant as the completely resistant *fre* mutant, but by 45 min, it became almost as sensitive as the *katEG* mutant (Fig. 3*B*). Since H_2_O_2_ levels remain constant in the *katEG fre* mutant (Fig. 3*A*), the initial resistance of this mutant compared to the *katEG* (Fre+) strain must be due to the absence of iron cycling. Then, the eventual H_2_O_2_-alone toxicity in the *katEG fre* mutant must have a different nature—for example, because of a sudden availability of Fe(II) from an earlier unavailable source or a switch to a different mode of killing. In fact, the pattern of complete initial resistance with an eventual deep killing was reminiscent of the delayed mode-two killing of WT cells by 25 mM H_2_O_2_ in the presence of deferoxamine (DF)+NO (22).

These two possibilities could be distinguished by chelating iron: additional iron recruitment should be blocked by DF, while mode-two killing should be insensitive to DF. DF addition rescued, albeit partially, the late sensitivity of the *katEG fre* mutant to H_2_O_2_-alone (Fig. 3*C*), meaning that both explanations apply to the late toxicity of H_2_O_2_ in the *katEG fre* mutant. Another evidence consistent with mode-two killing comes from chromosomal fragmentation: in contrast to the *katEG* mutant, where H_2_O_2_-alone induces CCF, fragmentation in the *katEG fre* mutant is much reduced (Fig. 3, *D* and *E*). Between 10 and 30 min, there is a loss of 20% intact chromosomal DNA, but no associated loss in viability. However, between 30 and 45 min, another 20% loss in chromosomal DNA results in loss in viability by three orders of magnitude. Thus, the killing of the *katEG fre* mutant by H_2_O_2_-alone cannot be explained by only double-strand DNA breaks.

Similar to the *fre* single mutant, which is partially resistant to the H_2_O_2_+NO treatment, the *katEG fre* triple mutant is initially resistant to H_2_O_2_+NO, but after 30 min again loses viability to reach survival titers similar to the *katEG* mutant (Fig. 3*F*). The same timing of viability loss in the *katEG fre* mutant during H_2_O_2_-alone (Fig. 3*B*) *versus* H_2_O_2_+NO (Fig. 3*F*) treatments indicates a significant metabolic switch in this mutant after 30 min of H_2_O_2_ exposure. In general, the overall similarity of the two sensitivity patterns means no further NO potentiation in the *katEG fre* triple mutant. This not only confirms that catalases are targets of NO inhibition but also implies that Fre controls an independent NO-potentiation pathway. Iron chelators completely save the *katEG fre* mutant from H_2_O_2_+NO treatment (Fig. 3*C*), again showing that NO blocks an unknown target of mode-two killing by H_2_O_2_. The chromosomal fragmentation and DNA disappearance during H_2_O_2_+NO treatment is again reduced in the *katEG fre* mutant relative to the *katEG* mutant (Fig. 3, *G* and *H*). Moreover, at 45 min there is again no significant increase in either fragmentation or DNA loss to explain the precipitous loss in viability at this late time point (Fig. 3*F*).

Thus, iron reduction offers at least two candidate activities, FtnA and Fre, for NO potentiation of H_2_O_2_ toxicity (Fig. 3*I*), as illustrated by the fact that minimizing iron reduction with the *fre* defect decreases drastically the density of double-strand breaks in the *katEG fre* mutant cells, saving them during the first 30 min of H_2_O_2_-alone or H_2_O_2_+NO treatments. In addition, the two-fold reduction of IF-iron in the NO-treated cells (Fig. 1, *H* and *I*) further elevates the importance of procurement of Fe(II) for H_2_O_2_+NO killing.

### Conditions for potentiation of H_2_O_2_ toxicity in the katEG mutants

In principle, NO pathways to potentiate H_2_O_2_ toxicity other than catalase inhibition could be revealed if lower bacteriostatic H_2_O_2_ concentrations for the *katEG* mutant could be again potentiated by NO. However, the static H_2_O_2_ concentrations for the *katEG* mutant in the range of 0.25 to 0.5 mM are not potentiated with 0.6 mM NO (Fig. S4*A*), implying that catalases are the only NO targets in our standard treatment conditions. Nevertheless, we found that lower NO concentrations, in the range of 0.06 to 0.15 mM, do sensitize the *katEG* mutant to 0.5 mM H_2_O_2_ treatment (Fig. S4, *B* and *C*), indicating existence of secondary NO targets. Moreover, blocking Fe(III)→Fe(II) reduction in the *katEG* mutant by the *fre* defect all but eliminates the sensitivity of the triple mutant to this milder 0.5 H_2_O_2_ + 0.06 NO treatment (Fig. S4*C*), suggesting that the potentiation of the secondary NO targets is still *via* Fe(II) generation fueling multiple Fenton cycles. In fact, in the previous paper, we reported that, in contrast to H_2_O_2_-alone treatment, H_2_O_2_+NO-treated *katEG* mutant is completely saved by iron chelation with DF (22). Potentiation by lower NO concentrations, but not by higher ones, show that in the *katEG* mutants, higher concentrations of NO inhibit, rather than potentiate, H_2_O_2_ toxicity, perhaps because excess NO acts as an iron chelator (Fig. 1, *H* and *I*).

Although we did find new NO-potentiating conditions for the *katEG* mutant (Fig. S4*B*), we decided to investigate the role of iron reduction in the chronic H_2_O_2_ toxicity with a gene candidate approach instead, using the standard conditions (2.5 mM H_2_O_2_ + 0.6 mM DEA NONOate), in order to align our readouts with previous results and to be able to also use catalase-proficient strains. Our objective was to verify the general metabolic process powering the FtnA/Fre-dependent IF-iron source that sustains Fenton in NO-treated cells. There was a strong candidate, as both iron recruitment from ferritins (46, 70) and iron reduction by Fre (44, 47) are promoted by reduced free flavins, like FMNs and FADH_2_ (Fig. 3*I*). So, how are the free flavins reduced?

### Respiration is exquisitely sensitive to NO

**Figure 4:**
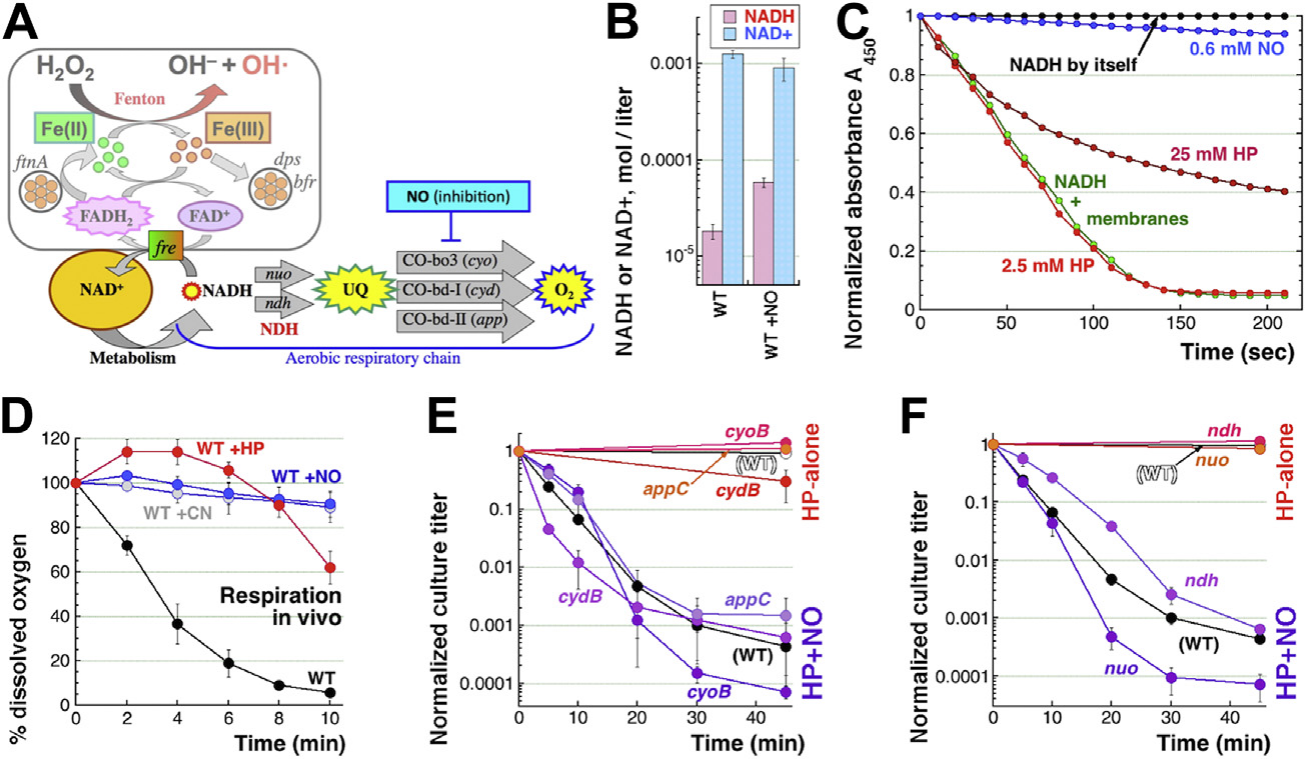
**Mutating individual enzymes of the aerobic respiratory chain does not change NO potentiation.***A*, a scheme of the electron transport (*yellow symbols*) and its connection to iron cycling *via* Fre. The upper-left part of the scheme corresponding to Figure. 3*I* is faded within the *gray* frame. NO should inhibit the three ubiquinol oxidases (CO, for cytochrome oxidase) catalyzing the final step. Inactivation of both NADH dehydrogenases (NDHs) should similarly lead to NADH accumulation and increased iron reduction. *B*, the cytoplasmic NADH and NAD^+^ concentration in WT cells, untreated or treated with 0.6 mM DEA NONOate for 5 min. *C, in vitro* NADH oxidation as a readout for respiration by inverted membrane vesicles shows no impact of 2.5 mM H_2_O_2_ treatment but ∼50% inhibition after 25 mM H_2_O_2_ treatment and almost complete inhibition by 0.6 mM NO. *D*, *in vivo* respiration by live cells, measured as the level of dissolved oxygen. Treatment with 3 mM CN or 60 μM NO inhibits it "permanently", while 0.25 mM H_2_O_2_ treatment appears to inhibit it transiently. *E,* H_2_O_2_-alone and H_2_O_2_+NO sensitivity of single mutants in the three cytochrome oxidases. *F,* H_2_O_2_-alone and H_2_O_2_+NO sensitivity of single mutants in the NDH NDHs. Fre, flavin reductase; H_2_O_2_/HP, hydrogen peroxide; NO, nitric oxide.

Woodmansee and Imlay proposed that the immediate target of NO inhibition is the electron transport chain of the aerobic respiration (40), specifically the heme-containing ubiquinol oxidases Cyo, Cyd, and App (Fig. 4*A*). The resulting NO-mediated respiratory block leads to accumulation of NADH, which in stably growing cells comprises a few percent of the total NAD pool (71), and this NADH excess facilitates reduction of FAD to FADH_2_ exactly by the same Fre, that also happens to reduce Fe(III) to the Fenton-catalyzing Fe(II), using reduced flavins as electron donors (Fig. 4*A*) (38, 40). Indeed, we detected a 3-fold increase in the absolute intracellular NADH concentrations in WT cells upon NO treatment (Fig. 4*B*), which translates into 4.5× increase of NADH fraction in the total NAD pools (Fig. S5). In the *in vitro* respiration assay, while 2.5 mM H_2_O_2_ does not affect NADH oxidation by inverted membrane vesicles, 600 μM DEA NONOate completely inhibits NADH oxidation (= respiration) (Fig. 4*C*). Respiration *in vivo* is measured as cellular oxygen consumption (depletion of oxygen from the chamber containing the oxygen electrode), where plunging oxygen levels indicate normal respiration (Fig. 4*D*, the black curve), while stable oxygen levels indicate inhibited respiration. Respiration in WT *E. coli* is expectedly inhibited by 3 mM CN and, unexpectedly and transiently, by a mere 0.25 mM H_2_O_2_ (Fig. 4*D*), the latter being a peculiar artefact of oxygen production by the catalase reaction (Fig. S6*A*). Indeed, respiration is unaffected by 0.25 mM H_2_O_2_ in the *katEG* mutant, even though higher H_2_O_2_ concentrations start inhibiting it in this mutant (Fig. S6*B*), probably reflecting mode-two killing (22). This might also explain why the *katEG fre* mutant shows a period of resistance to H_2_O_2_-alone, as H_2_O_2_ mimics NO’s respiration inhibition in the *katEG* mutants. Although we could not challenge *in vivo* respiration with 600 μM DEO NONOate due to technical issues, we found that even 60 μM DEO NONOate inhibits this *in vivo* respiration completely (Fig. 4*D*), showing exquisite sensitivity of ubiquinol oxidases to NO (we will return to this point later).

**Figure 5:**
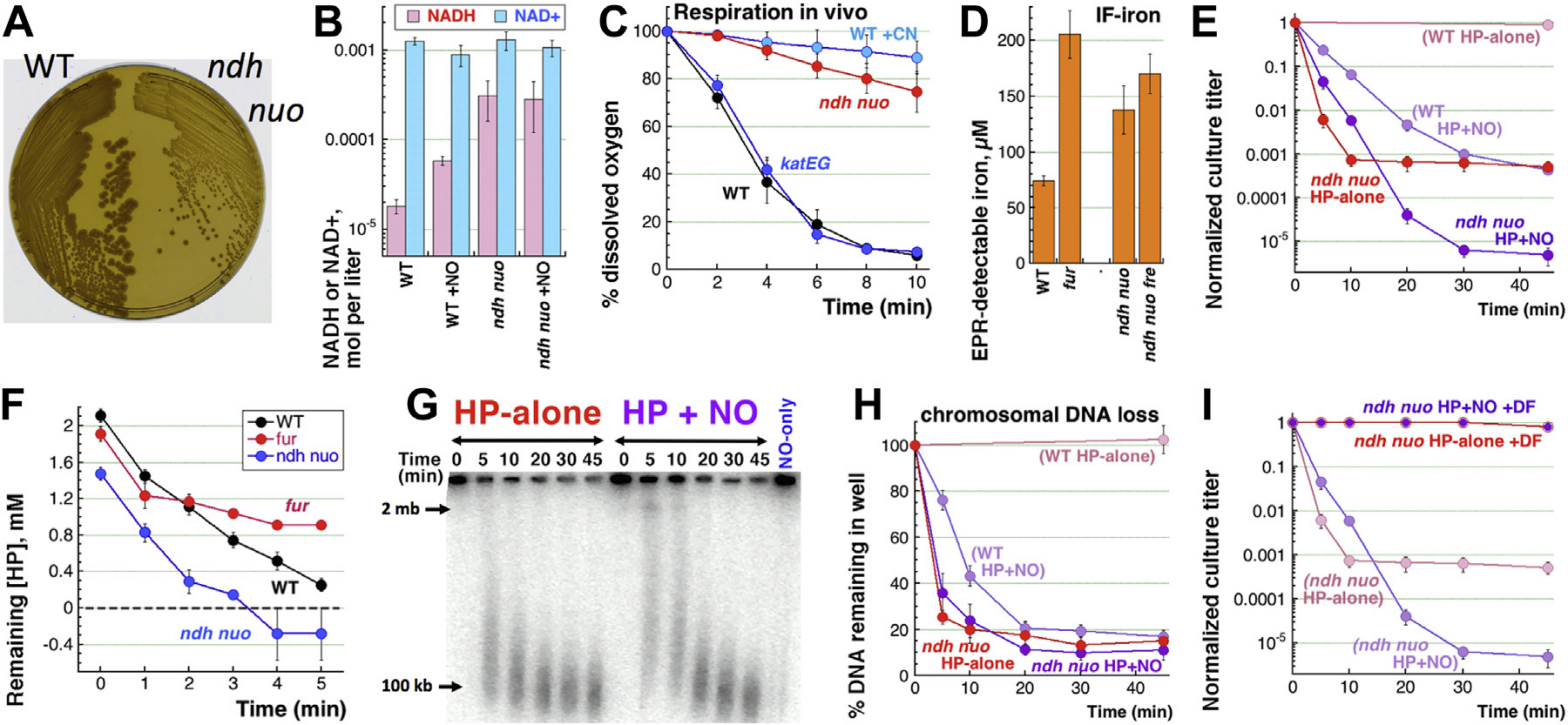
**Disruption of the aerobic respiratory chain sensitizes *E. coli* to H**_**2**_**O**_**2**_**.***A*, colony size of the *ndh nuo* mutant compared to the WT. *B*, the cytoplasmic NADH and NAD^+^ concentration in the *ndh nuo* mutant cells, treated or not with 0.6 mM DEA NONOate for 5 min. The WT results from Figure. 4*B* are shown for comparison. *C,* the level of residual oxygen consumption in the *ndh nuo* mutant. *D,* IF-iron levels in the *ndh nuo* and *ndh nuo fre* mutants. WT and *fur* data are shown as controls. *E,* H_2_O_2_-alone and H_2_O_2_+NO sensitivity of the *ndh nuo* double mutant. *F,* H_2_O_2_ disappearance in the *ndh nuo* mutant cultures compared with WT and *fur* cultures. *G,* a representative gel illustrating chromosome fragmentation patterns caused by H_2_O_2_-alone or H_2_O_2_+NO treatments in the *ndh nuo* double mutant. *H,* quantification of the chromosomal DNA loss in the *ndh nuo* double mutant in response to H_2_O_2_-alone or H_2_O_2_+NO treatments, from several gels like in "G". *I,* both H_2_O_2_-alone and H_2_O_2_+NO toxicity in the *ndh nuo* double mutant are blocked by DF. DF, deferoxamine; EPR, electron paramagnetic resonance; H_2_O_2_/HP, hydrogen peroxide; IF-iron, intracellular free iron; NO, nitric oxide.

As already mentioned, there are three ubiquinol oxidases in *E. coli,* Cyo, Cyd, and App, active during aerobic/oxic respiration (Fig. 4*A*)*.* We found that eliminating any one of the three individual ubiquinol oxidases has no phenotype, in that the *cyoB*, or *cydB,* or *appC* single mutants are completely resistant to H_2_O_2_-alone treatment, while showing WT-like sensitivity to the H_2_O_2_+NO treatment (Fig. 4*E*). However, the double mutants in any two of the three cytochrome oxidases made the mutant growth impractically slow, so we decided to block the electron transport chain one step earlier, at the level of NADH dehydrogenases (NDH), Nuo and Ndh (Fig. 4*A*). Single *ndh* or *nuo* mutants again showed WT resistance to H_2_O_2_-alone and similar to WT sensitivity to H_2_O_2_+NO treatment (Fig. 4*F*) suggesting redundancy of the two enzymes under our growth conditions. It was the mutant lacking both NDHs that should be unable to respire and was expected to accumulate NADH, boosting the Fe(III)→Fe(II) reduction and thus sensitizing cells to H_2_O_2_. The *ndh nuo* double mutant is viable, even though slow-growing (Fig. 5*A*); the mutant indeed accumulates ∼10 times more NADH compared to the WT cells, and this NADH level does not further respond to the NO treatment (Figs. 5*B* and S5), suggesting that it is already at the maximum. If the NADH level is a critical indicator, the *ndh nuo* mutant should show significantly stronger H_2_O_2_ toxicity effects than those induced in WT cells by NO treatment.

### The *ndh nuo* mutants are extremely sensitive to H_2_O_2_-alone

While WT cells consume O_2_ within 8 min, the *ndh nuo* mutant utilizes little O_2_ to respire aerobically, similar to the control respiration-inhibited WT cells treated with 3 mM CN (Fig. 5*C*). The IF-iron is increased about 2-fold in the *ndh nuo* mutant, to the levels between the WT and the *fur* mutant (Fig. 5*D*). The *ndh nuo* double mutant was extremely sensitive to H_2_O_2_-alone, dying ∼3 times faster than WT cells during H_2_O_2_+NO treatment, but eventually to the same final level of survival of ∼10^−3^ (Fig. 5*E*). The sudden plateauing of the survival by 10 min is because the *ndh nuo* mutant scavenges H_2_O_2_ faster than WT cells, within 3 min (Fig. 5*F*). The only catalase-proficient mutants known to be (slightly) sensitive to H_2_O_2_-alone were *fur* (Fig. 2*A*) and *sodAB* (Fig. 1*G*) (21), likely because of IF-iron overload in these mutants (Fig. 1, *H* and *I*) (66), so the higher IF-iron in the *ndh nuo* mutant supports this correlation. However, a major part of its high sensitivity to H_2_O_2_-alone is likely due to the higher NADH levels (Fig. 5*B*) promoting iron reduction.

Addition of NO exacerbated the H_2_O_2_ lethality of *ndh nuo* double after 20 min of treatment so that, by 45 min, survival became 10^-5^ (Fig. 5*E*). At the same time, in the first 20 min, NO slowed down H_2_O_2_ killing of the *ndh nuo* double, although the rate was still faster than in the WT cells (Fig. 5*E*). These two opposite effects created a peculiar "hybrid" sensitivity curve, with "NO-protection" during the first 20 min switching to NO potentiation after 20 min (Fig. 5*E*). Since the *ndh nuo* double mutant is killed by both H_2_O_2_-alone and H_2_O_2_+NO treatments, this generally confirms the previous idea (38, 40) that block of the respiratory chain is a way NO could potentiate H_2_O_2_ toxicity. However, the shapes of H_2_O_2_-alone *versus* H_2_O_2_+NO sensitivity curves in the *ndh nuo* mutant are sufficiently different (Fig. 5*E*) to suggest that NO changes the nature of H_2_O_2_ toxicity in the *ndh nuo* mutant, probably by complexing IF-iron, as reflected by its decrease in NO-treated cells (Fig. 1, *H* and *I*).

While 2.5 mM H_2_O_2_-alone fails to cause fragmentation in WT cells (21, 22), it induces speedy CCF in the *ndh nuo* mutant (Fig. 5, *G* and *H*); moreover, the kinetics of CCF induced in the *ndh nuo* mutant by H_2_O_2_+NO appears slower (Fig. 5, *G* and *H*), again suggesting that NO actually retards the precipitous H_2_O_2_-alone toxicity in this mutant. Both the killing by H_2_O_2_-alone and by H_2_O_2_+NO in the *ndh nuo* mutant are blocked by iron chelation with DF (Fig. 5*I*), indicating exclusively (iron-dependent, chromosomal DNA-targeting) mode-one killing. The blocking effect of DF confirms that respiration inhibition poisons cells *via* increased iron reduction.

Limiting iron reduction with the *fre* defect makes the *ndh nuo fre* triple mutant significantly less sensitive to the H_2_O_2_-alone killing (Fig. S7), demonstrating that a significant part of the *ndh nuo* mutant sensitivity to H_2_O_2_-alone is due to iron cycling, rather than simply due to the elevated IF-iron. At the same time, *ndh nuo fre* mutant’s IF-iron levels are similarly elevated (Fig. 5*D*), implying that the residual sensitivity of these mutants to H_2_O_2_-alone is primarily due to the higher initial IF-iron levels. In short, our investigation reveals some correlation between initial IF-iron levels and sensitivity to H_2_O_2_ but also demonstrates that the rate of iron reduction and the H_2_O_2_ stability are more important for the *in vivo* effects of Fenton chemistry.

### Interaction between the *ndh nuo* and *katEG* defects

**Figure 6:**
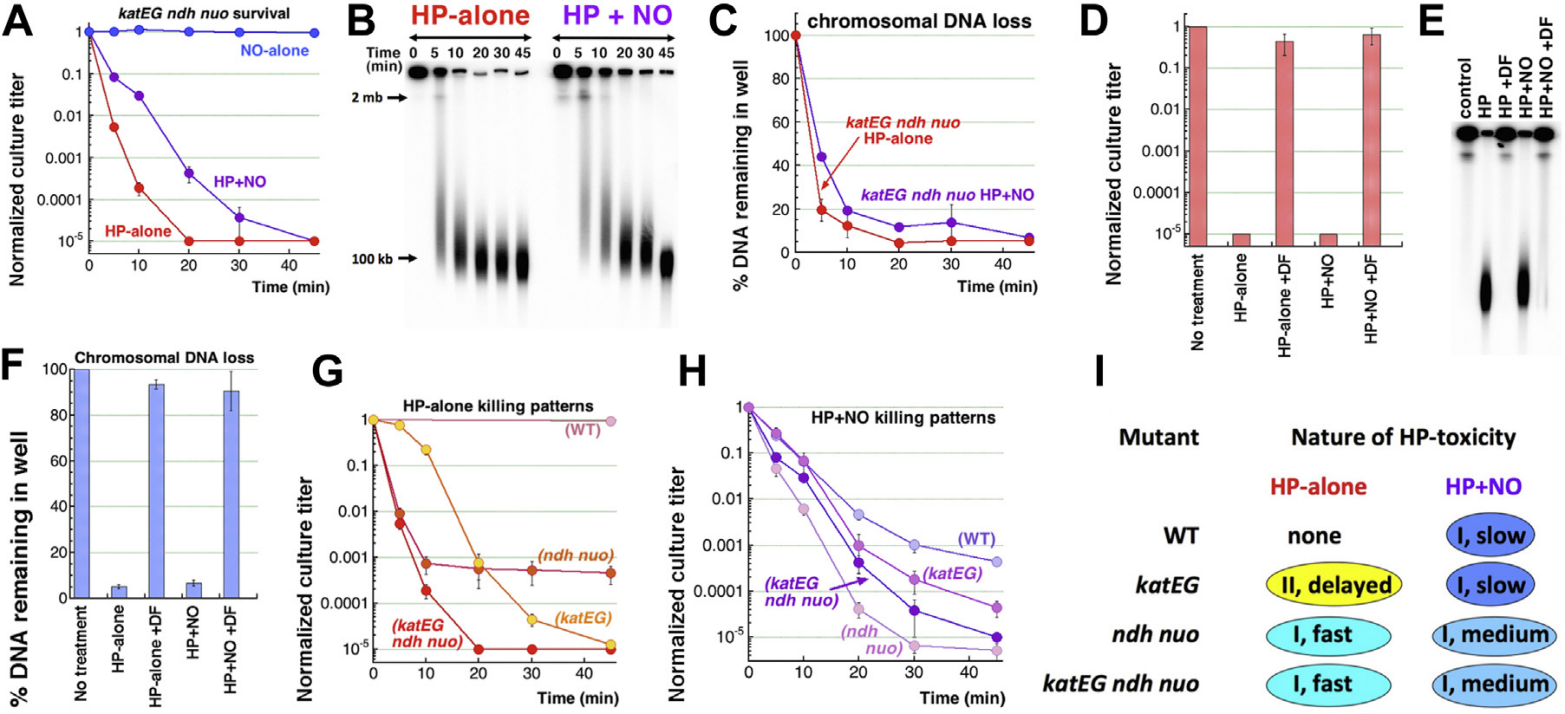
**NO slows down the H**_**2**_**O**_**2**_**killing of the *katEG ndh nuo* quadruple mutant.***A*, H_2_O_2_-alone and H_2_O_2_+NO sensitivity of the quadruple *katEG ndh nuo* mutant. *B*, a representative gel illustrating chromosome fragmentation patterns caused by H_2_O_2_-alone and H_2_O_2_+NO treatments in the *katEG ndh nuo* quadruple mutant. *C,* quantification of the chromosomal DNA loss in the *katEG ndh nuo* quadruple mutant in response to H_2_O_2_-alone or H_2_O_2_+NO treatments, from several gels like in "B". *D,* the effect of iron chelation with DF on H_2_O_2_-alone or H_2_O_2_+NO sensitivity of the *katEG ndh nuo* mutant. *E,* a representative gel illustrating the effect of iron chelation with DF on the chromosome fragmentation in the *katEG ndh nuo* mutant treated with either H_2_O_2_-alone or with H_2_O_2_+NO for 45 min. *F,* quantification of the chromosomal DNA loss from several gels like in "E". *G,* comparison of H_2_O_2_-alone killing patterns of the WT, *katEG* double, *ndh nuo* double, and the *katEG ndh nuo* quadruple mutants. *H,* comparison of H_2_O_2_+NO killing patterns of the WT, *katEG* double, *ndh nuo* double, and the *katEG ndh nuo* quadruple mutants. *I*, qualitative parameters of 2.5 mM H_2_O_2_ toxicity of H_2_O_2_-alone *versus* H_2_O_2_+NO treatments in various mutants. *I,* mode-one killing; II, mode-two killing. DF, deferoxamine; H_2_O_2_/HP, hydrogen peroxide; NO, nitric oxide.

Thus, we have characterized two double mutants for their chromosome fragmentation and viability: (1) the catalase-deficient *katEG* mutant (22) and (2) the NDH–deficient *ndh nuo* mutant (Fig. 5). Both combinations render *E. coli* cells sensitive to H_2_O_2_-alone treatments, but for opposite reasons in terms of the Fenton reactants: H_2_O_2_ stability in *katEG versus* Fe(II) flow in *ndh nuo*. In order to genetically test whether the *ndh nuo versus* the *katEG* defects sensitize cells to H_2_O_2_-alone by distinct pathways or the same pathway, we constructed a *katEG ndh nuo* quadruple mutant, in which increased iron cycling is combined with H_2_O_2_ stability. The quadruple mutant was not sensitive to NO-alone, showed a somewhat higher sensitivity to H_2_O_2_+NO, and was exquisitely sensitive to H_2_O_2_-alone treatment (Fig. 6*A*). This sensitivity of the quadruple mutant reflected the extreme rates of chromosome fragmentation after either treatment (Fig. 6, *B* and *C*). Iron chelation with DF completely suppressed the sensitivity of the quadruple mutant to both H_2_O_2_-alone or H_2_O_2_+NO (Fig. 6*D*), as well as the CCF induced by both treatments (Fig. 6, *E* and *F*), indicating that this fast killing is strictly mode-one. In other words, the unknown targets of H_2_O_2_ mode-two killing in the *katEG* mutant (22) are efficiently masked by the *ndh nuo* defect.

Comparison of the kinetics of H_2_O_2_-alone and H_2_O_2_+NO sensitivity curves of the four strains, WT, *katEG, ndh nuo,* and *katEG ndh nuo,* proved insightful. For the H_2_O_2_-alone treatment, to which WT cells were completely resistant, the effect of two pairs of H_2_O_2_-sensitizing mutations turned out to be additive. As a result, the quadruple mutant responded to the H_2_O_2_-alone treatment with a composite sensitivity curve, in which the early (fast) killing aspect of the *ndh nuo* mutant was combined with the late (deep) killing effect of the *katEG* mutant (Fig. 6*G*). Apparently, the early killing was due to the increased iron cycling and IF-iron levels (reflecting the *ndh nuo* defect), while the continuous later killing was due to the stability of H_2_O_2_ (reflecting the *katEG* defect). We conclude that *ndh nuo* and the *katEG* represent two independent pathways protecting *E. coli* against acute H_2_O_2_ toxicity.

This point was further corroborated by H_2_O_2_+NO treatment of the *katEG ndh nuo* quadruple mutant; compared with H_2_O_2_-alone, we observed ∼2.5-fold slower rate of killing (Fig. 6*A*), the effect that we have already observed with *ndh nuo* double mutant (Fig. 5*B*). In other words, even though NO potentiates H_2_O_2_ toxicity in WT cells, it clearly protects some H_2_O_2_-hypersensitive mutants from the same H_2_O_2_ concentrations. As a result of this "NO-buffering", in contrast to the different kinetics of killing of WT, *katEG, ndh nuo,* and *katEG ndh nuo* strains with H_2_O_2_-alone (Fig. 6*G*), the same four strains treated with H_2_O_2_+NO show, somewhat counterintuitively, similar initial rates ending with depth of killing differences within two orders of magnitude (Fig. 6*H*). We conclude that the nature of H_2_O_2_ toxicity, though masked by cellular resistance mechanisms in WT cells, is complex (mode-one + mode-two), while NO potentiation amplifies H_2_O_2_ toxicity even in WT cells, but also makes it mechanistically simpler, converting it to a slower mode-one in all mutant combinations (Fig. 6*I*).

### Catalases and the respiratory chain are the major targets of NO in *E. coli*

**Figure 7:**
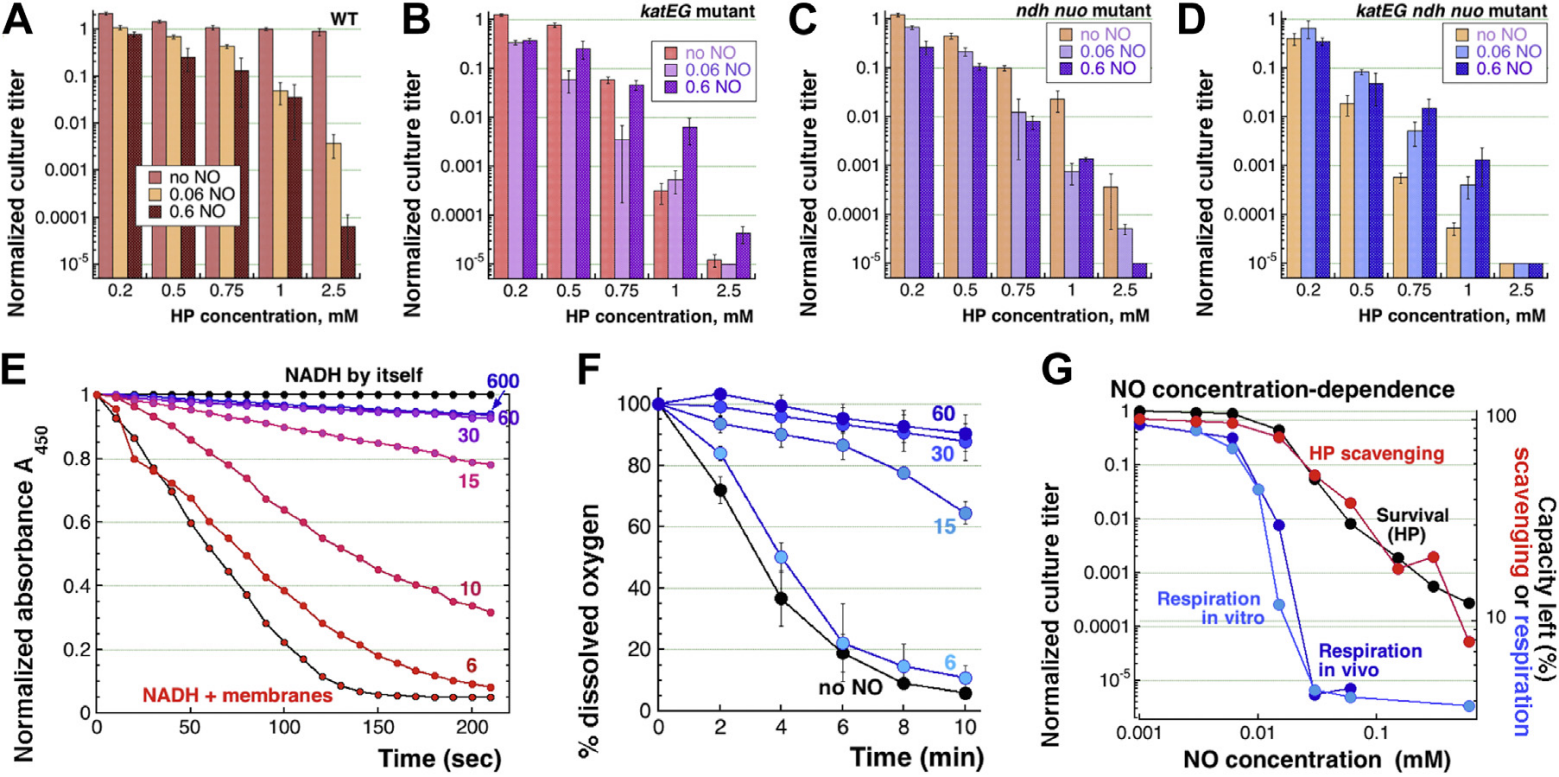
**NO fails to potentiate H**_**2**_**O**_**2**_**toxicity in the *katEG ndh nuo* quadruple mutant.** All the treatments were for 45 min. *A*, H_2_O_2_-alone up to 2.5 mM is not toxic to WT cells but becomes toxic (in any concentration) in the presence of 0.6 mM NO and if ≥ 1 mM, even in the presence of 0.06 mM NO. *B*, the H_2_O_2_-alone toxicity in the *katEG* double mutant can be potentiated at the intermediate H_2_O_2_ concentrations with 0.06 mM NO. *C*, the H_2_O_2_-alone toxicity in the *ndh nuo* double mutant is potentiated at any H_2_O_2_ concentrations with both 0.06 mM and 0.6 mM NO. *D,* both NO concentrations always alleviate toxicity of the intermediate H_2_O_2_ concentrations in the *katEG ndh nuo* quadruple mutant. *E,* concentration dependence of the NO inhibition of *in vitro* respiration (NADH oxidation by inverted membrane vesicles). Individual curves are marked by DEA NONOate concentrations (μM). *F,* concentration dependence of the NO inhibition of *in vivo* respiration (oxygen consumption). Individual curves are marked by DEA NONOate concentrations (μM). *G,* comparison of survival of H_2_O_2_+NO-treated WT cells at various [NO] with the degree of remaining "capacity", either for respiration (measured at 2 min in panel "E", at 10 min in panel "F") or for H_2_O_2_ scavenging (22), at the same range of [NO]. Error bars are omitted for clarity. H_2_O_2_/HP, hydrogen peroxide; NO, nitric oxide.

In order to reveal the remaining pathways of NO potentiation of H_2_O_2_ toxicity, if any, we treated the *katEG ndh nuo* quadruple mutant with varying concentrations of H_2_O_2_-alone, looking for residual NO targets that potentiate H_2_O_2_ toxicity with two DEA NONOate concentrations, 0.06 mM and 0.6 mM (Fig. 7*D*). As controls, we tested WT, *katEG* double mutant, and *ndh nuo* double mutant with the same treatments as the quadruple mutant (Fig. 7, *A–C*). As shown previously (22), WT cells are not sensitive to any H_2_O_2_-alone concentration in this range, from 0.2 to 2.5 mM, while gradually increasing potentiation with NO from low [H_2_O_2_] to high [H_2_O_2_] and with higher [NO] potentiating better (Fig. 7*A*). In contrast, in the *katEG* mutant, 0.75 mM, 0.5 mM, and 0.2 mM H_2_O_2_ can be potentiated by 0.06 mM NO (corroborating Fig. S4 results), while the higher 0.6 mM NO concentration has no effect at these H_2_O_2_ concentrations and even starts protecting *katEG* mutants at the highest H_2_O_2_ concentrations (Fig. 7*B*). This was earlier interpreted to mean mode-two killing in the *katEG* mutant, with NO switching it to mode-one, slower killing (Fig. 6*I*) (22). In the *ndh nuo* mutant (Fig. 7*C*), H_2_O_2_ was lethal by itself and was further potentiated by both 0.6 mM and 0.06 mM NO, similar to WT cells (compared to Fig. 7*A*). Finally, in the *katEG ndh nuo* mutant, the lethality of the three intermediate H_2_O_2_ concentrations was reduced by both NO concentrations (Fig. 7*D*), suggesting no more targets for H_2_O_2_ potentiation by NO in this mutant of *E. coli*.

The lack of NO potentiation of the higher [H_2_O_2_] in the *katEG* mutant (Fig. 7*B*), the remaining ability to potentiate these [H_2_O_2_] with NO in the *ndh nuo* mutant (Fig. 7*C*) and finally, the disappearance of this potentiation with the removal of catalases in the *katEG ndh nuo* mutant (Fig. 7*D*) suggest that catalase inhibition by NO is a major cause of lethality during the 2.5 mM H_2_O_2_ + 0.6 mM DEO NONOate treatment. We suspected that this was because respiration, the other NO potentiation route, was more sensitive to lower NO concentrations that were still not enough to potentiate H_2_O_2_ toxicity. To test this idea, we measured respiration inhibition, both *in vitro* (Fig. 7*E*) and *in vivo* (Fig. 7*F*), by various low concentrations of NO and found that both processes are completely inhibited by 30 μM DEA NONOate (Fig. 7, *E* and *F*), the concentration that inhibits catalases by less than 50% and causes ∼one order of magnitude of H_2_O_2_ killing (22).

In other words, [NO]-dependence of respiration inhibition does not explain continued H_2_O_2_ killing at NO concentrations higher than 30 μM. To illustrate this, we plotted inhibition of respiration *versus* H_2_O_2_ scavenging at various concentrations of NO with the corresponding killing curve by H_2_O_2_+NO (again at various concentrations of the latter) (Fig. 7*G*). In general, the shapes of the two curves were different enough to conclude that respiration inhibition cannot be the main reason for H_2_O_2_+NO lethality at the millimolar H_2_O_2_ concentrations used in this study. In particular, 15 μM of DEA NONOate was sufficient to inhibit 90% of respiration *in vivo*, while this NO exposure still was not enough to cause lethality in H_2_O_2_-treated cells (Fig. 7*G*). At the same time, as shown previously, the shape of H_2_O_2_ scavenging inhibition by various [NO] coincided with the H_2_O_2_+NO killing curve (Fig. 7*G*), strongly suggesting that it is the former that drives the latter (22).

## Discussion

We have previously systematically examined the earlier observations (32, 40) that NO potentiates toxicity of otherwise static concentrations of H_2_O_2_, so the latter becomes lethal for WT *E. coli* by continually inducing multiple double-strand DNA breaks, which lead to catastrophic fragmentation of the chromosome the cells cannot repair (22). We also showed that a major NO potentiation pathway of H_2_O_2_ toxicity is by inhibition of heme-containing catalases, making H_2_O_2_ levels stable and thus enabling continuous Fenton chemistry with IF-iron. Since the *katEG* catalase–deficient mutant was equally sensitive to H_2_O_2_+NO and H_2_O_2_-alone treatments, catalase inhibition appeared to provide adequate explanation for NO potentiation (22). However, this answer could not be complete, as Fenton, in addition to H_2_O_2_, also requires Fe(II), yet H_2_O_2_ entry into the cytoplasm will (presumably) instantaneously oxidize all IF-Fe(II) to Fe(III), self-limiting Fenton's damage. In addition, Dps mini-ferritin will use H_2_O_2_ to sequester all remaining IF-iron; however, these two challenges notwithstanding, NO somehow ensures a flow of Fe(II) even in the presence of H_2_O_2_, making Fenton continuous.

This work explored the nature of this continuous source of Fe(II) in NO-treated cells. We found that a potential system maintaining the pool of reduced iron in H_2_O_2_-treated cells are the iron depots ferritins (FtnA), along with the previously identified Fre, and continuous free flavin reduction supported by the increased pools of NADH, resulting from the respiration block by NO. We modeled NO-inhibition of ubiquinol oxidases by genetically blocking the preceding step of NADH oxidation in the *ndh nuo* double mutant, deficient in NDH activity. The *ndh nuo* mutant not only accumulates NADH but also has increased IF-iron and is killed by H_2_O_2_-alone even faster than by H_2_O_2_+NO, confirming respiration inhibition as another route of NO potentiation of H_2_O_2_ toxicity. The *katEG ndh nuo* quadruple mutant, that keeps both the H_2_O_2_ and Fe(II) levels high, shows a remarkable sensitivity to H_2_O_2_-alone and instead of being potentiated by NO is actually saved by it, demonstrating that NO potentiation pathways are exhausted in the mutant. Finally, we show that respiration is inhibited at low NO concentrations, at which little lethality in the H_2_O_2_+NO treatments is observed. In contrast, lethality correlates well with catalase inhibition, which happens gradually and over a range of higher NO concentrations, elaborating our previous conclusion about H_2_O_2_ stability by complementing it with the nature of a continuous source of Fe(II).

### Progress since previous studies

Imlay and Linn had shown some time ago that CN makes static concentrations of H_2_O_2_ lethal (23, 72). Based on their previous work, Woodmansee and Imlay argued that CN has no effect on the intracellular H_2_O_2_ concentrations; they also showed that CN increases the IF-iron only two times and therefore argued that CN makes *E. coli* sensitive to low mM H_2_O_2_ *via* inhibition of respiration, by producing electron donor that drives the Fenton reaction (38). Using semiquantitative PCR, they reported NO enhancement of *in vivo* DNA damage by H_2_O_2_. They also tested *in vitro* whether accumulation of NADH is directly responsible for Fe(III) reduction and had to reject this idea; eventually they found that the proximal Fe(III) reductant is a free flavin, FADH_2_, produced by Fre, in the reaction driven by NADH (38). The authors then extended their observations to potentiation of H_2_O_2_ toxicity with NO, again linking it to the increased DNA damage *in vivo* (40). In particular, they documented inactivation of Fe-S cluster enzymes, but no iron release from them; in fact, in their study, NO-alone treatment reduced IF-iron in the treated cells in half, just like in our case (Fig. 1, *H* and *I*). They showed that *cyo cyd* double mutant (the one with significant growth defects to be usable under our conditions) is extremely sensitive to H_2_O_2_-alone treatment and that NO fails to increase this sensitivity further (40), suggesting that ubiquinol oxidases of the respiratory chain are the targets of NO inhibition.

Our previous studies (21, 22) and the current one complement and extend their findings in several ways: (1) by demonstrating that catalases are also targets of NO inhibition, and their inactivation guarantees a stable presence of H_2_O_2_ for continuous Fenton; (2) by proposing that both CN and NO recruit additional iron from FtnA ferritin, *via* the same Fre-driven reduction dependent on the elevated NADH levels, while mini-ferritin Dps and bacterioferritin Bfr sequester iron during H_2_O_2_ treatment; (3) our *ndh nuo* double mutant behaves similar to their *cyo cyd* double mutant (extreme sensitivity to H_2_O_2_-alone, with no additional sensitization by NO), confirming the importance of respiratory chain in providing reduced iron for Fenton; (4) that no additional NO targets, beyond catalases and respiratory terminal oxidases, contribute to NO potentiation of H_2_O_2_ toxicity; and (5) last but not least, that DNA damage during the H_2_O_2_+NO or H_2_O_2_+CN treatments takes the form of CCF (breaking the chromosome into at least 100 pieces), explaining why the cells cannot repair it.

Separately, the H_2_O_2_+NO sensitivity of the *bfr* mutant, especially in the *bfr dps* double mutant combination, appears to be the first time that *E. coli bfr* mutant shows any phenotype, in contrast to the strong iron-accumulation defects of the *bfr* mutants in *Pseudomonas*, for example (15). One of the functions of bacterioferritin in *E. coli*, therefore, is to sequester IF-iron in conditions of oxidative stress—similar to the Dps function in *E. coli* or to the Bfr function in anaerobe *Desufovibrio* (73).

### Preexisting Fe(II) IF-iron levels versus Fe(III) reduction during the treatment

The deep, CCF-based lethality of oxidative damage is remarkable if we consider that its nature is based on H_2_O_2_ simply rising to certain concentrations in the cytoplasm of the affected cells and interacting with IF-iron to produce a burst of hydroxyl radicals. Indeed (1) the known low concentration of (presumably) dispersed IF-iron should produce enough OH· for only limited damage to DNA, because the oxidative impact will be similarly dispersed around the cytoplasm; (2) the resulting DNA damage should be all single-stranded (mostly nicks, but also some base lesions) (74); (3) the time course of the DNA damage should be brief, restricted to the first few minutes of the treatment. Basically, Fenton driven only by IF-iron should be self-limiting. Some chromosomal damage is demonstrably driven by IF-iron levels during the first few minutes of H_2_O_2_-alone treatment, as illustrated by the initial dip in viability and detectable fragmentation after H_2_O_2_-alone treatment of the *fur* and *sodAB* mutants (Figs. 1*G*, 2, *A* and *C*) (21), which have higher IF-iron concentrations. However, if IF-iron level was the most important factor, then the *ndh nuo* mutant, with its IF-iron level lower than that of *fur* mutant (Fig. 5*D*), would show better survival and less fragmentation after H_2_O_2_-alone treatment. In contrast to this expectation, H_2_O_2_-alone treatment kills the *ndh nuo* mutant fast and deep (Fig. 5*E*), apparently because of the rapid fragmentation of its chromosomal DNA (Fig. 5, *G* and *H*). Thus, DNA damage is mostly driven by a reason other than the preexisting levels of Fe(II) IF-iron.

This reason, apparently, is continuous Fe(III) reduction to Fe(II), supported by high levels of NADH in the *ndh nuo* mutants and the elevated NADH levels in the NO-treated cells (Fig. 5*B*). This continuous source of Fe(II) Fenton reactant explains not only the observed massive DNA damage but also why Fenton in H_2_O_2_+NO-treated cells is not self-limiting. Indeed, in the H_2_O_2_+NO-treated cells, fragmentation still continues 1 hour later, indicating that it depends not only on the stability of H_2_O_2_ in the presence of NO but also on the continuous source of Fe(II). An additional evidence for the importance of iron cycling over IF-iron levels is offered by the *ndh nuo fre* mutant, which has the same levels of IF-iron as its *ndh nuo* progenitor (Fig. 5*D*) but is much less sensitive to H_2_O_2_-alone treatment (Fig. S7), because of the *fre* defect in Fe(III) reduction.

Accumulation of NADH in the *ndh nuo* mutant suggests that rapid NADH oxidation by the electron transport chain in WT cells provides an effective shield against oxidative damage, while its inactivation puts Fenton chemistry in overdrive. It is also interesting to note that genetic inactivation of the *ndh nuo* pathway does more than to simply phenocopy NO-treatment. This is apparent for both WT background (compare WT H_2_O_2_+NO *versus ndh nuo* H_2_O_2_-alone in Fig. 5*E*) and in the *katEG* background (compare *katEG* H_2_O_2_+NO of Fig. 6*H* *versus katEG ndh nuo* H_2_O_2_-alone of Fig. 6*G*). The obvious explanation for the differences is that NO (at least at 0.6 mM DEA NONOate) does not cause the same level of NADH accumulation as the *ndh nuo* inactivation (Fig. 5*B*)—and thus, the lower expected level of Fe(III) reduction. Perhaps there are minor ubiquinol oxidases (not inhibited by NO?) in *E. coli* yet to be characterized?

### The IF-iron versus Fenton-active iron

Since Fre and ferritin FtnA are both important for the lethality of the H_2_O_2_ + NO treatment in WT cells and since Fe(III), because of its higher charge, was expected to form complexes tighter than Fe(II) with big molecules like DNA (75), we expected to see increased IF-iron (= Fe(II)) in WT cells treated with NO. In fact, both NO and reduced flavins have previously been shown to release iron from ferritins *in vitro* (44, 46, 47, 76). However, our EPR analysis detected less iron in WT cells treated with NO than in untreated cells (Fig. 1*H*), as was also reported by others (40). Therefore, in addition to iron release from ferritins and reduction by Fre, NO promotes iron removal from IF-iron pool, a complexing of a kind, that also enhances DNA damage. What is the nature of this removal?

The only way to increase DNA damage from Fenton without dramatically increasing the overall Fenton in the cell would be to run Fenton in the vicinity of DNA, preferably on DNA itself. Therefore, it was proposed that NO not only induces release of iron from ferritins and promotes its reduction by Fre but also recruits this iron to DNA, both removing it from IF-iron pool and increasing the DNA-damaging potential of subsequent Fenton (31, 72). We have observed a similar scenario with CN *in vitro*; although CN forms stable complexes with free iron, when plasmid DNA is added, the iron–CN still binds this DNA, causing plasmid nicking in the presence of H_2_O_2_ (21). It would be interesting to repeat these *in vitro* experiments with NO.

### Protection by NO

Not only does NO potentiate H_2_O_2_ toxicity but also its mode of action reverses to protection against H_2_O_2_ toxicity under certain conditions. For example, others showed that NO protected *B. subtilis* from H_2_O_2_ by limiting Fenton and recharging catalase (54, 77). As explained in the introduction, there are two distinct modes of H_2_O_2_ toxicity, DNA-targeting iron-dependent mode-one *versus* iron-independent mode-two with unknown target. We observed that while iron chelators cannot save *katEG* mutants from mode II toxicity of H_2_O_2_, there is complete survival with the same H_2_O_2_ treatment when NO is additionally present (22). In other words, NO can function as an iron chelator and, in effect, helps other chelators to shield IF-iron from H_2_O_2_. In contrast to the *katEG* mutant, killed by mode-two with 2.5 mM H_2_O_2_, the same H_2_O_2_ concentration kills the *katEG ndh nuo* mutant by mode-one (Fig. 6*D*), implying the mode-two target is gone in the absence of NDHs. While the *katEG* mutants grow using aerobic respiration (Fig. 5*C*), the NDH mutants, *ndh nuo* and *katEG ndh nuo*, do not respire (Fig. 5*C*) and likely grow fermentatively. Thus, NO mimics the *ndh nuo* mutations and inhibits the fast mode-two killing, apparently by binding and protecting an undetermined target in the respiratory chain.

It isn’t only in the presence of chelators that NO shows its defensive side. Since NO targets both catalases and respiration, it could be expected conservatively that the loss in viability in the *katEG ndh nuo* mutant with H_2_O_2_ will be similar to that observed with H_2_O_2_+NO in WT cells (compare Fig. 7, *A* and *D*). However, the H_2_O_2_ lethality in the mutant is much higher than the H_2_O_2_+NO lethality in WT, revealing protective effects of NO in the WT cells. This is observed more clearly in the *katEG ndh nuo* survival of H_2_O_2_ challenge, where NO slows down cell killing considerably (Fig. 7, *A* and *D*). In general, NO's effects in the cell vary, explaining contrasting effects of its combination with H_2_O_2_ in various mutants. We posit the two general ways NO could protect against H_2_O_2_ toxicity: (i) NO blocks a mode II target related to aerobic respiration (for example by binding heme iron) and (ii) NO acts as a general weak iron chelator.

### Peroxynitrite

Since phagosomes produce H_2_O_2_ indirectly, *via* superoxide (59), the simultaneous production of NO necessitates the discussion of the potential contribution of peroxynitrite (ONOO-), which rapidly forms in reaction between NO and superoxide (61). Because of its ability to spontaneously yield hydroxyl radicals (78), peroxynitrite is proposed to be the toxic species behind the bactericidal power of macrophages (3). However, since it is the protonated form of peroxynitrite that preferentially penetrates the bacterial cell envelope (while the charged ONOO– has to use anion channels) and with its pKa close to neutral, peroxynitrite becomes really poisonous for *E. coli* at pH significantly higher than physiological ones (60), but its increased instability at these pH negates its toxicity (33). Moreover, acute peroxynitrite treatment of *E. coli* fails to induce the SOS response, suggesting no significant DNA damage but instead induces transcriptional responses pointing to protein nitration and nitrosylation as the main cytoplasmic impact (79). This is inconsistent with the idea that H_2_O_2_+NO treatment acts *via* peroxynitrite, as this combined treatment is notable for its DNA damaging power (22, 32, 40). Finally, the idea that H_2_O_2_+NO treatments works *via* generating peroxynitrite around bacterial cells is inconsistent with the lack of protection against the treatment by bicarbonate, which completely protects against bona fide peroxynitrite (33).

In our experimental system, peroxynitrite contribution to the overall H_2_O_2_+NO toxicity could be only minor, for the following reasons: (1) were peroxynitrite a major contributor, the superoxide dismutase-deficient *sodAB* mutant would be more sensitive to H_2_O_2_+NO treatment, but in fact it is more resistant than WT (Fig. 1*G*); (2) peroxynitrite is toxic independently of iron (60, 78), whereas NO-promoted toxicity of H_2_O_2_ is blocked by iron chelation (22, 40); (3) if inhibition of respiration by NO indeed generated enough superoxide, then NO-alone treatment *via* formation of peroxynitrite inside cells would at least affect WT cells and would kill the *sodAB* mutants—but it does not (Fig. 1, *A* and *G*). Further experiments are needed to clarify any potential role of peroxynitrite formation in NO-potentiated H_2_O_2_ toxicity and its underlying chromosome fragmentation.

## Conclusion

NO potentiates the intracellular Fenton reaction, causing lethality *via* CCF. NO potentiation has two major routes, and both occur *via* its binding to heme-containing enzymes: (i) inhibition of catalases to make H_2_O_2_ stable and (ii) inhibition of respiration to boost iron recruitment and reduction in the presence of H_2_O_2_. In the future, it would be important to develop conditions with similar effects but utilizing more physiological low micromolar concentrations of H_2_O_2_ and NO. Due to its polyanionic nature, DNA binds iron avidly, creating a natural platform for Fenton chemistry. The resulting hydroxyl radicals should induce singly damaged sites including nicks in DNA, but their relationship to double-strand DNA breaks that fragment the chromosome is still unclear. Finally, it would be interesting to explore the interactions between ferritins and DNA in the presence of H_2_O_2_ and NO *in vitro* using plasmid-nicking assays.

## Experimental procedures

### Strains and plasmids

Our *E. coli* strains (Table S1) are all derivatives of K-12 BW25117 (80). Alleles were moved between strains by P1 transduction (81). The mutants were all deletion-replacements from the Keio collection, purchased from the *E. coli* Genetic Stock Center and all verified by PCR (and also phenotypically, whenever possible).

### Enzymes and reagents

Catalase from bovine liver, H_2_O_2_, deferoxamine mesylate, horseradish peroxidase, and o-dianisidine:2HCl were all purchased from Sigma. DEA-NONOate was from Cayman Chemical. A 60 mM stock solution of DEA-NONOate was prepared fresh each time by dissolving several milligrams of the chemical in 0.1 M NaOH. NAD^+^/NADH assay kit was from Abcam (ab65348).

### Growth conditions and viability assay

To generate killing kinetics, fresh overnight cultures were diluted 1000-fold into modified lysogeny broth (LB) [10 g tryptone, 5 g yeast extract, 5 g NaCl, 250 μl 4 M NaOH per liter (81), buffered with 50 mM Tris-HCl (pH 8.0) (the "LB8" medium)] (22). The stabilization of pH was required for reproducibility of NO delivery by DEA NONOate. Cultures were shaken at 37 °C for about 3 h or until they reached exponential phase (A_600_ ∼ 0.3). At this point, the cultures were made 0.6 mM for DEA NONOate and/or 2.5 mM for H_2_O_2_ (these two standard concentrations were used throughout the experiments; nonstandard concentrations are specified in a few experiments), and the shaking at 37 °C was continued. Viability of cultures was measured at the indicated time points by spotting 10 μl of serial dilutions in 1% NaCl on LB plates (LB medium above supplemented with 15 g of agar per liter). The plates were developed overnight at 28 °C, and the next morning, the pin-prick colonies in each spot with 10 to 200 colonies were counted under the stereomicroscope. All titers were normalized to the titer at time 0 (before addition of the treatment). For the iron chelator treatment, cultures grown as above were made 20 mM for deferoxamine mesylate 5 minutes before hydrogen peroxide treatment.

### Measurement of relative H_2_O_2_ concentrations

This follows our previous protocol (22). The 40 mM o-dianisidine stock preparation: 318 mg of o-dianisidine:2HCl was added to 10 ml of 95% ethanol, then mixed with 25 ml of DI water. Assay cocktail: 60 μg/ml horseradish peroxidase, 150 μM o-dianisidine in potassium phosphate buffer (50 mM KP_i_, 0.1 mM EDTA, pH 7.8), kept ice-cold. Overnight cultures were diluted 1000-fold into LB8 medium and shaken for 3 h at 37 °C (A_600_ ∼ 0.3). At the desired timepoint after addition of H_2_O_2_ (±NO), 300 μl aliquots of cultures were withdrawn and cleared of the cells in a microcentrifuge for 1 min. Culture supernatant was diluted 1:10 into the potassium phosphate buffer. The diluted sample (667 μl) was mixed with 333 μl of the assay cocktail, and after 45 s at room temperature (∼20 °C), absorbance at 460 nm was measured.

### Measuring chromosomal fragmentation by pulsed-field gel electrophoresis

This generally follows our previous protocols (82, 83). All strains were grown in LB8 medium; overnight cultures were diluted 1000-fold and grown with 1 to 10 μCi of ^32^P-orthophosphoric acid per milliliter of culture for 3 h at 37 °C (*A*_600_ ∼ 0.3) before addition of 0.6 mM NO + 2.5 mM H_2_O_2_ (or the indicated treatment). The reactions were stopped by addition of 312 μg of catalase (13 μl of 24 mg/ml stock), and aliquots of the culture were taken at the indicated times to make plugs. Cells of the aliquot were spun down, resuspended in 60 μl of TE buffer, and put at 37 °C. A total of 2.5 μl of proteinase K (5 mg/ml) was added, immediately followed by 65 μl of molten 1.2% agarose in the lysis buffer (1% sarcosine, 50 mM Tris HCl pH 8.0, and 25 mM EDTA) held at 70 °C. The mixture was pipetted a couple of times before being poured into a plug mold and let solidify for 2 min at room temperature. The plugs were then pushed out of the molds and incubated overnight at 60 °C in 1 ml of the lysis buffer. Half-plugs were loaded into a 1.0% agarose gel in 0.5× Tris–borate–EDTA buffer and run at 6.0 V/cm with the initial and the final switch times of 60 and 120 s, respectively, at 12 °C in CHEF-DR II PFGE system (Bio-Rad) for 20 to 22 h. The gel was vacuum dried at 80 °C (on Whatman paper) and then exposed to a PhosphorImager screen (Fujifilm) overnight. The resulting signals were quantified with a PhosphorImager (Fuji Film FLA-3000).

### Electrochemical detection of NO and H_2_O_2_

Actual concentrations of NO and H_2_O_2_ were measured using NO sensor ISO-NOP (22, 84) and H_2_O_2_ sensor ISO-HPO2 connected to the TBR4100 Free Radical Analyzer (World Precision Instruments). Before calibration, the sensors were polarized in PBS (137 mM NaCl, 2.7 mM KCl,10 mM Na_2_HPO_4_, 1.8 mM KH_2_PO_4_, pH 7.4) for over 12 h and 2 h, respectively. The NO sensor was calibrated by adding increasing concentrations of KNO_2_ to 0.1 M H_2_SO_4_+0.1 M KI. Changes in current (ΔpA) corresponding to increasing NO concentrations were measured to generate a standard curve. To measure NO released from DEA NONOate, LB or LB8 media were incubated at 37 °C on a temperature probe–controlled heated stir plate with stirring set at 170 rpm. The baseline current was recorded before DEA NONOate was added to the desired concentration, and the new current was recorded. The ΔpA calculated by subtracting baseline was used to determine the actual concentrations of NO. The H_2_O_2_ sensor was calibrated by adding increasing concentrations of H_2_O_2_ to PBS. Changes in current (ΔpA) corresponding to increasing H_2_O_2_ concentrations were measured to generate a standard curve (Fig. S2*A*). To measure H_2_O_2_ in cultures, 300 μl aliquots were withdrawn and cleared of the cells in a microcentrifuge for 1 min. The baseline current in PBS was recorded. Culture supernatant was diluted 1:10 into PBS, and the new current was recorded. The ΔpA calculated by subtracting baseline was used to determine the actual concentrations of H_2_O_2_.

### O_2_ consumption assay

Cells were cultured to *A*_600_ = 0.2 in LB8 as described above. Respiration was measured with a Digital Model 10 Clark-type oxygen electrode (Rank Brothers, Ltd) at 37 °C, as described before (85, 86). NO, H_2_O_2_, or CN were added to the desired concentrations, once the oxygen electrode chamber was filled and equilibrated with cell culture. The machine was calibrated by air-saturated LB medium and sodium dithionite.

### NADH consumption assay

This was done as described (85). Overnight cultures in LB8 supplemented with 0.2% glucose were diluted to *A*_600_ = 0.010 in 1 L LB8 with 0.2% glucose and were grown to A_600_ = 0.3. Cells were collected by centrifugation for 5 min at 10,000*g*, resuspended in 10 ml ice-cold potassium phosphate buffer (50 mM, pH 7.8), and lysed with French press. After pelleting debris by spinning for 20 min at 20,000*g*, supernatant was spun further for 2 h at 140,000*g* to collect the membrane pellet. Membranes were resuspended in 3 ml ice-cold potassium phosphate buffer by repeated pipetting and stored on ice at 4 °C. The NADH consumption assay was performed with 200 μM NADH and 20 μl membranes. After addition of CN or nitric oxide, when needed, the total reaction volume was made 1000 μl with potassium phosphate buffer.

### NAD^+^/NADH measurement assay

Extracts from *E. coli* cells were prepared and processed as described (87). Briefly, overnight LB8 cultures were diluted to *A*_600_ = 0.003 in 40 ml LB8 and grown to *A*_600_ = 0.25 to 0.3 at 37 °C with shaking. When appropriate, cells were treated with 0.6 mM DEA NONOate (final concentration) for 5 min. Cells were collected by filtration and resuspended in 700 μl ice-cold extraction buffer (from the Abcam kit). For NAD extraction, the resuspended cells were further diluted 50-fold in extraction buffer, lysed with 0.2 M HCl at 55 °C for 10 min, and neutralized to pH∼7.0 with NaOH. For NADH measurement, in the *ndh nuo* mutant, the filtered and resuspended cells were further diluted 10-fold in extraction buffer. For WT and NO-treated cells, the filtered and resuspended cells were used directly. Cells were lysed instantly with 0.2 M NaOH at 55 °C for 10 min and neutralized to pH∼7.0 with HCl. Cells were centrifuged at 12,000 rpm for 3 min to remove debris, and the collected supernatant was used directly in the Abcam NAD^+^/NADH assay protocol.

### IF-iron measurements

The procedure generally follows the protocol by Sen *et al.* (48) with some differences. Cells were grown in 500 ml LB8 to A_600_ between 0.1 and 0.25. When appropriate, cells were made 0.6 mM for DEA NONOate or 3 mM for CN and incubated for 10 min. Cells were harvested by centrifugation at 7000*g* for 5 min at 4 °C. The cell pellet was resuspended in 10 ml LB8 prewarmed to 37 °C. The medium also contained 10 mM DETAPAC (diethylenetriaminepentaacetic acid, pH 7.0) and 20 mM DF (pH 8.0). The cells were incubated at 37 °C for 15 min with shaking at 220 rpm. The cells were washed twice with 2 ml of ice-cold 20 mM Tris-HCl pH 7.4 and then resuspended in 300 μl of ice-cold 30% glycerol, 20 mM Tris-HCl pH 7.4, transferred into an EPR tube and frozen on dry ice with ethanol. The A_600_ of the final cell suspension was measured after a 1:1000 dilution. Ferric chloride standards were prepared in the same Tris buffer containing glycerol. The spectrometer settings were the following: microwave power, 10 mW; microwave frequency, 9.05 GHz; modulation amplitude, 12.5 G at 100 KHz; time constant, 0.032; temperature, 15°K.

## Data availability

All data described in the manuscript are contained within the manuscript itself.

## Supporting information

This article contains supporting information (22, 80, 88).

## Conflict of interest

The authors declare that they have no conflicts of interest with the contents of this article.
