# Supplementary material for: Nitric oxide precipitates catastrophic chromosome fragmentation by bolstering both hydrogen peroxide and Fe(II) Fenton reactants in E. coli
Source: J Biol Chem. 2022 Mar 11;298(4):101825. doi: 10.1016/j.jbc.2022.101825 (PMC9018393; doi:10.1016/j.jbc.2022.101825)
Supplement: Supplemental Figures S1–S7 and Table S1 [file mmc1.pdf]

Supplement for the paper

Nitric oxide bolsters both hydrogen peroxide and Fe(II) Fenton reactants in vivo, precipitating catastrophic chromosomal fragmentation in *E. coli*

by

Pooja Agashe and Andrei Kuzminov

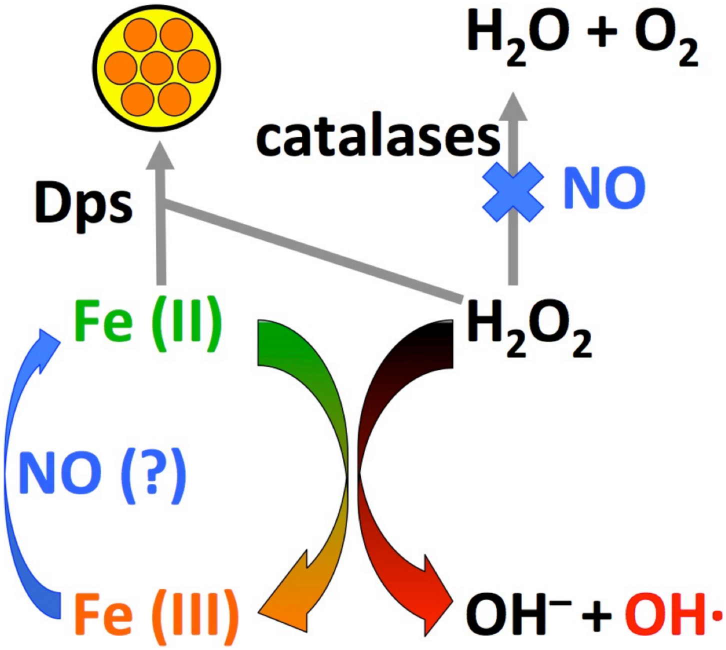

Figure S1. Fenton's reaction and two ways to boost it. The two substrates of Fenton are Fe(II) and H_2O_2. The cell prevents Fenton by: 1) packing Fe(II) as iron(III)-oxide into Dps spheres, using H_2O_2 as an oxidant; 2) removing H_2O_2 with catalases. One way NO boosts Fenton that we have characterized previously is via catalase inhibition (AGASHE AND KUZMINOV 2021), which leaves open the question about the source of Fe(II), which is actively removed, both by Dps and by Fenton itself. In this work we concentrate on possible ways for NO to ensure a constant source of Fe(II) in the presence of H_2O_2.

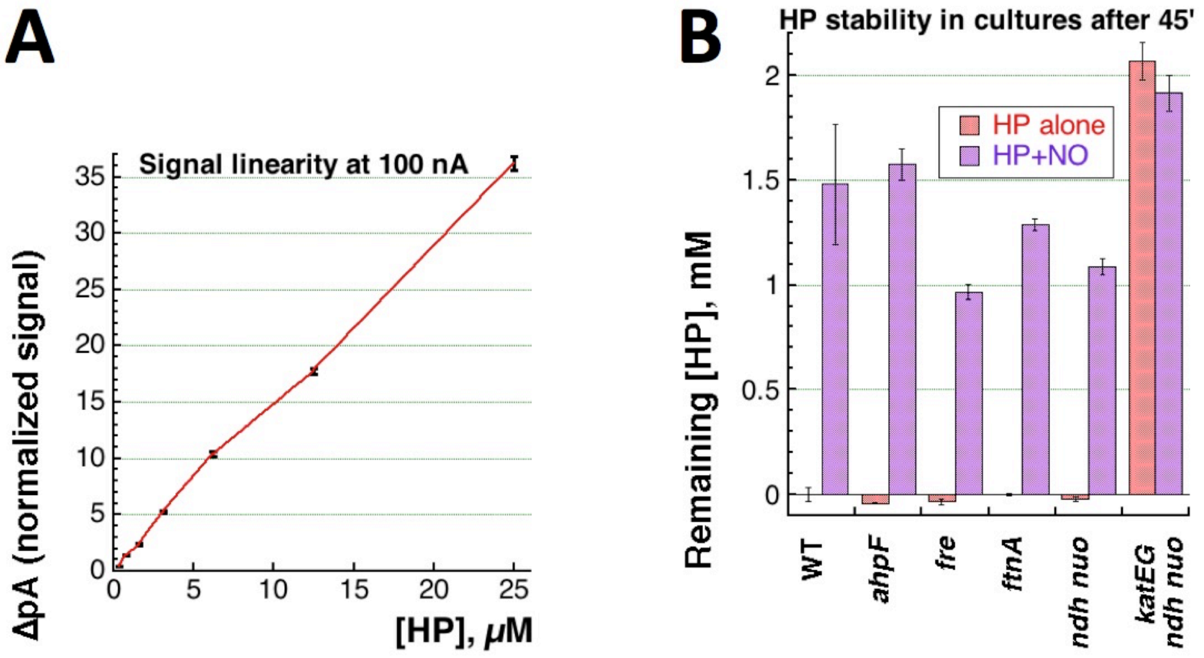

Fig. S2. H_2O_2 measurements with the H_2O_2 electrode.

A. The calibration curve with pure H_2O_2.

B. Stability (survival) of 2.5 mM H_2O_2 in suspension of the indicated mutants, either as H_2O_2-alone, or in mixtures of H_2O_2+NO.

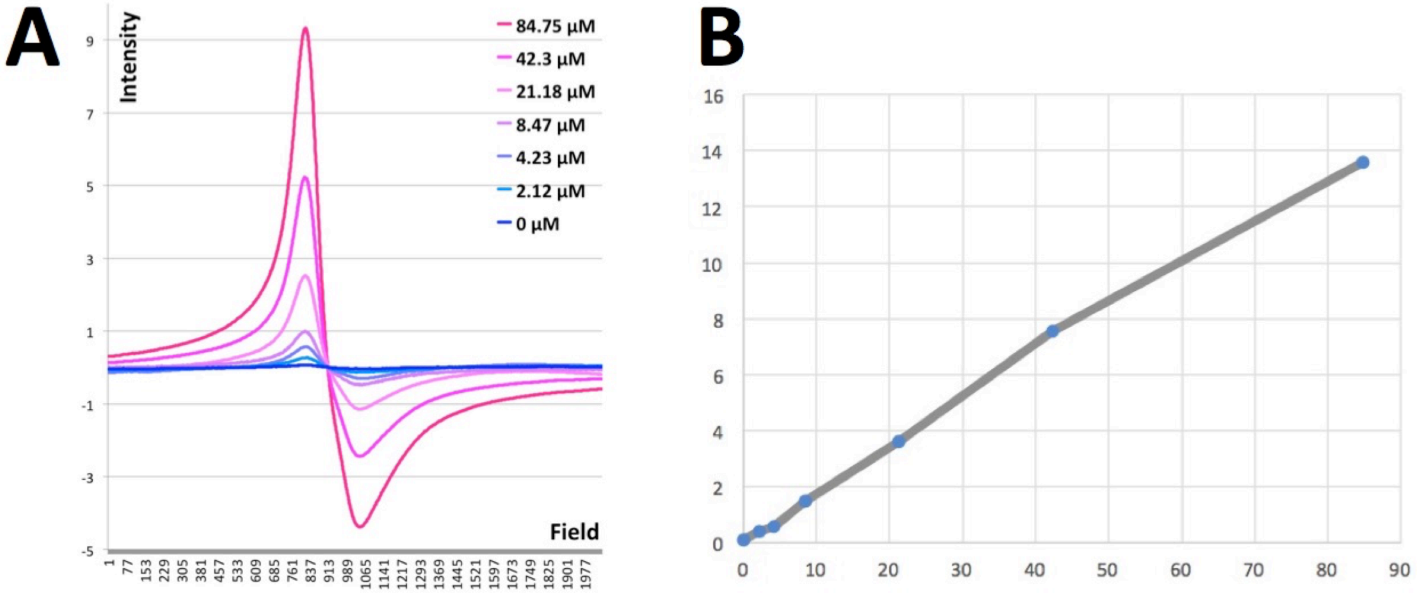

Fig. S3. EPR determination of iron amounts: building the standard curve.

A. The EPR signals from various known amounts of iron.

B. The standard curve itself. (X-axis: Fe concentration, Y-axis: Intensity)

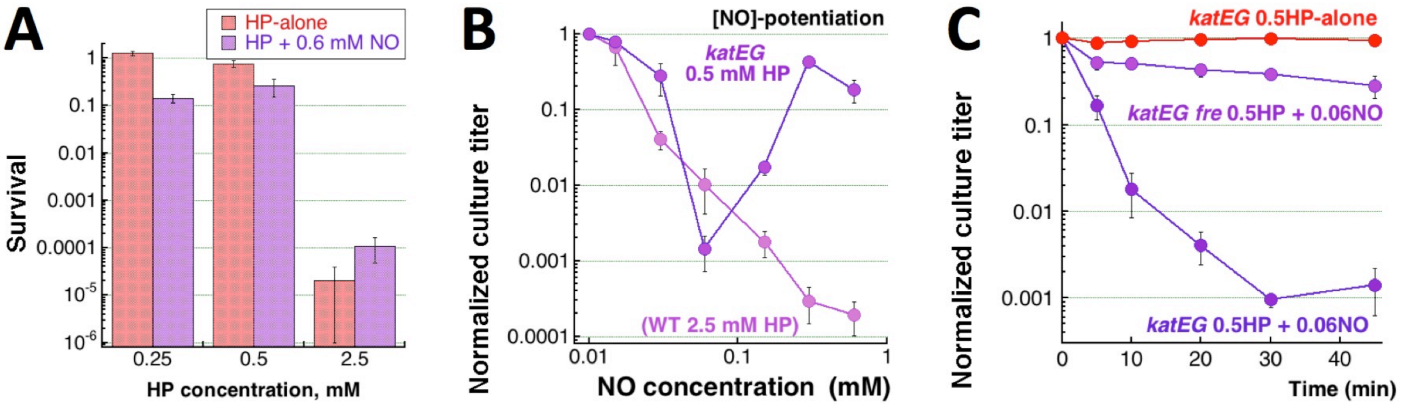

Fig. S4. NO still potentiates H_2O_2-toxicity in the *katEG* double mutant, at lower concentrations of both.

A. Negligible potentiation of H_2O_2 treatments of the *katEG* mutant by 0.6 mM NO.

B. Finding potentiating concentrations of NO. The treatment was for 45 minutes.

C. The *fre* defect still saves the *katEG* mutant from the NO-potentiated H_2O_2 toxicity.

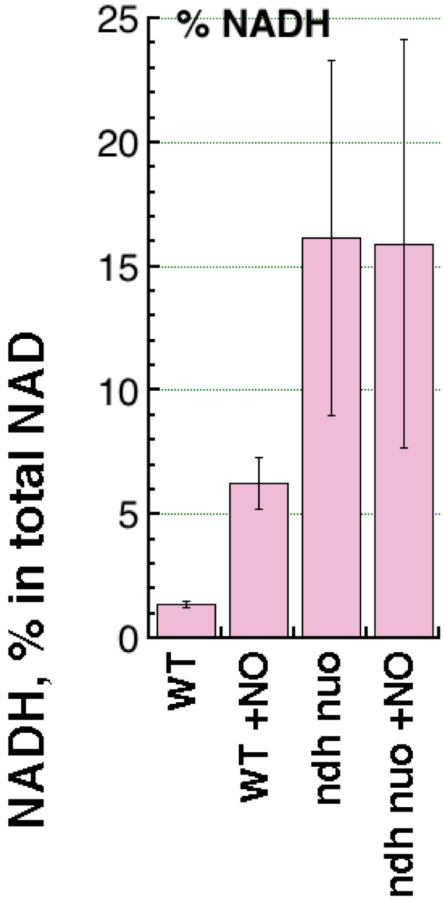

Fig. S5. The level of NADH as % of the total NAD pools. The NO-treatment was for 5 minutes.

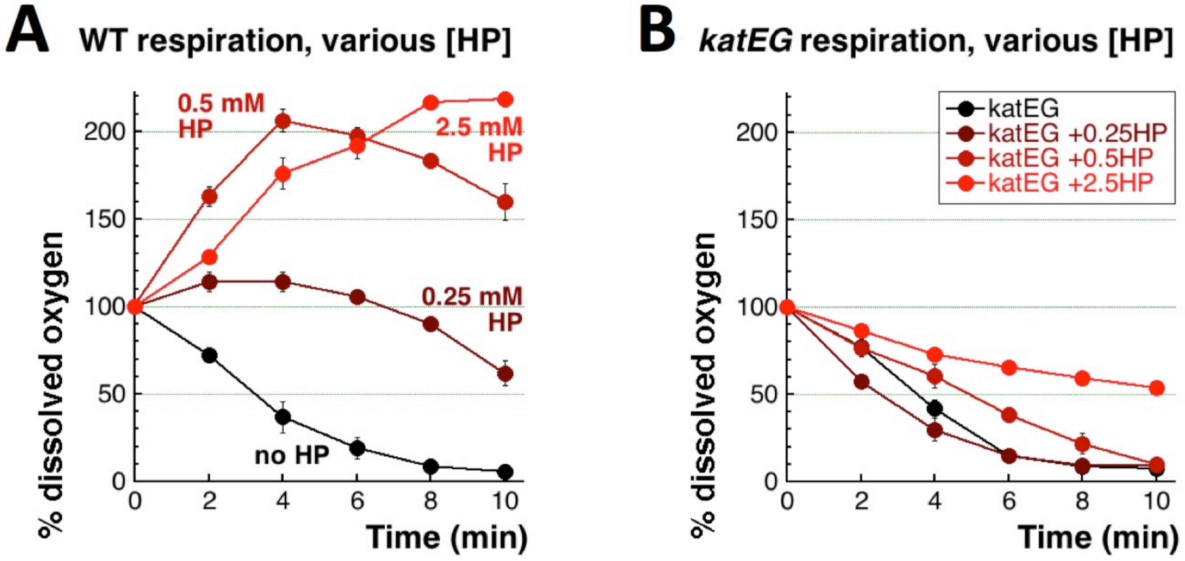

Fig. S6. Effect of H_2O_2 on in vivo respiration cannot be monitored by dissolved oxygen, because oxygen is produced by the catalase reaction: $2H_2O_2 \rightarrow 2H_2O + O_2$.

A. In vivo respiration (the level of dissolved oxygen) by WT cells in the presence of various [H_2O_2].

B. The same, but for the *katEG* mutant.

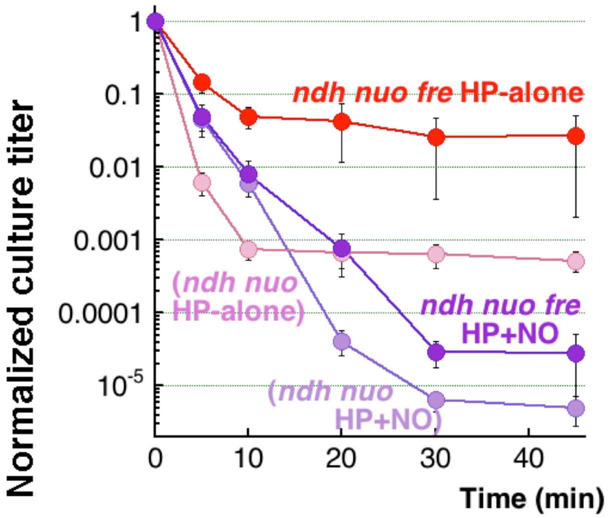

Fig. S7. H_2O_2**-alone and** H_2O_2**+NO sensitivity of the *ndh nuo fre* triple mutant.**

Table S1. E. coli strains.

Strain name	Relevant genotype	Source/CGSC# or Construction
BW25113	F-, Δ*(araD-araB)567*, Δ*lacZ4787*(::rrnB-3), λ^{-}, *rph-1*, Δ*(rhaD-rhaB)568*, *hsdR514*	7636, (BABA *et al.* 2006)
JW1721-1	Δ*katE731*::*kan*	9453
JW3914-1	Δ*katG729*::*kan*	10827
TM10	Δ*katE732*	JW1721-1 –> pCP20, 42°C, screen for Kn^S
TM12	Δ*katG729::kan* Δ*katE732*	TM10 x P1 JW3914-1
JW0669-2	Δ*fur-731::kan*	8758
JW3820-1	Δ*fre-784::kan*	10763
JW1893-1	Δ*ftnA-755::kan*	9575
JW0797-1	Δ*dps-784*::*kan*	8844
JW3298-1	Δ*bfr-746::kan*	10467
PA10	Δ*bfr-747*	JW3298-1–> pCP20, 42°C, screen for Kn^S
PA11	Δ*bfr-747* Δ*dps-784*::*kan*	PA10 X P1 JW0797-1
TM12	Δ*ftnA-756*	JW1893-1–> pCP20, 42°C, screen for Kn^S
TM15	Δ*ftnA-756* Δ*fre-784::kan*	TM12 x P1 JW3820-1
PA12	Δ*katG730* Δ*katE732*	TM12–> pCP20, 42°C, screen for Kn^S
PA13	Δ*katG730* Δ*katE732* Δ*fre-784::kan*	PA12 X P1 JW3820-1
CP909	*nuoG*::Tn*10 purF*	(PRÜSS *et al.* 1994)
TM17	*nuoG*::Tn*10 purF*	BW2113 x P1 CP909
JW1095-1	Δ*ndh-771::kan*	11791
PA14	Δ*ndh-771::kan nuoG*::Tn*10 purF*	JW1095-1 X P1 TM17
JW0960-1	Δ*appC721*::*kan*	8956
JW0421-1	Δ*cyoB788*::*kan*	8585
JW0723-2	Δ*cydB782*::*kan*	8790
PA15	*nuoG*::Tn*10 purF* Δ*ndh-772*	PA14–> pCP20, 42°C, screen for Kn^S
PA16	*nuoG*::Tn*10 purF* Δ*ndh-772* Δ*fre-784::kan*	PA15 X P1 JW3820-1
PA17	Δ*katG730* Δ*katE732* Δ*ndh-772:kan*	PA12 X P1 JW1095-1
PA18	Δ*katG730* Δ*katE732* Δ*ndh-772:kan nuoG*::Tn*10 purF*	PA17 X P1 TM17

References

Agashe, P., and A. Kuzminov, 2021 Catalase inhibition by nitric oxide potentiates hydrogen peroxide to trigger catastrophic chromosome fragmentation in *Escherichia coli*. Genetics 218**:** iyab057.

Baba, T., T. Ara, M. Hasegawa, Y. Takai, Y. Okumura *et al.*, 2006 Construction of *Escherichia coli* K-12 in-frame, single-gene knockout mutants: the Keio collection. Mol. Syst. Biol. 2**:** 2006.0008.

Prüss, B. M., J. M. Nelms, C. Park and A. J. Wolfe, 1994 Mutations in NADH:ubiquinone oxidoreductase of *Escherichia coli* affect growth on mixed amino acids. J. Bacteriol. 176**:** 2143-2150.
